# Characterization of *Ginkgo biloba* Leaf Flavonoids as Neuroexocytosis Regulators

**DOI:** 10.3390/molecules25081829

**Published:** 2020-04-17

**Authors:** Choongjin Ban, Joon-Bum Park, Sora Cho, Hye Rin Kim, Yong Joon Kim, Hyungjin Bae, Chinhan Kim, Hakhee Kang, Davin Jang, Yong Sub Shin, Dae-Ok Kim, Hyunggun Kim, Dae-Hyuk Kweon

**Affiliations:** 1 Institute of Biomolecule Control and Institute of Biologics, Sungkyunkwan University, Suwon 16419, Gyeonggi, Korea; pahncj@skku.edu; 2 Department of Integrative Biotechnology, Sungkyunkwan University, Suwon 16419, Gyeonggi, Korea; hybrik@skku.edu (J.-B.P.); hryrin@gmail.com (H.R.K.); vgbk491@skku.edu (Y.J.K.); 3 Interdisciplinary Program in BioCosmetics, Sungkyunkwan University, Suwon 16419, Gyeonggi, Korea; jsora7254@gmail.com; 4 C&I lab, Kolmar Korea Co., Ltd., Seoul 06792, Korea; hjbae@kolmar.co.kr (H.B.); kimch@kolmar.co.kr (C.K.); hhkang@kolmar.co.kr (H.K.); 5 Graduate School of Biotechnology, Kyung Hee University, Yongin 17104, Gyeonggi, Korea; davin1031@khu.ac.kr (D.J.); zipw4234@naver.com (Y.S.S.); 6 Department of Food Science and Biotechnology, Kyung Hee University, Yongin 17104, Gyeonggi, Korea; 7 Department of Biomechatronic Engineering, Sungkyunkwan University, Suwon 16419, Gyeonggi, Korea

**Keywords:** biocompatibility, bioactivity, flavonoids, *Ginkgo biloba* leaf isolate, neurotransmission inhibition, physicochemical stability, antioxidant activity, anti-inflammatory activity

## Abstract

*Ginkgo biloba* leaf (GBL) is known as a potential source of bioactive flavonoids, such as quercetin, arresting the neuronal soluble *N*-ethylmaleimide-sensitive factor attachment protein receptor (SNARE)-zippering. Here, the GBL flavonoids were isolated in two different manners and then examined for their bioactivity, physicochemical stability, and biocompatibility. The majority of flavonoids in the non-hydrolyzed and acidolyzed isolates, termed non-hydrolyzed isolate (NI) and acidolyzed isolate (AI) hereafter, were rich in flavonol glycosides and aglycones, respectively. Glycosidic/aglyconic quercetin and kaempferol were abundant in both NI and AI, whereas a little of apigenin, luteolin, and isorhamnetin were found in AI. NI was more thermostable in all pH ranges than quercetin, kaempferol, and AI. NI and AI both inhibited neurotransmitter release from differentiated neuronal PC-12 cells. NI and AI showed 1/2–1/3 lower EC_50_/CC_50_ values than quercetin and kaempferol. The NI and AI exhibited no toxicity assessed by the tests on chorioallantoic membranes of hen’s eggs, removing toxicological concerns of irritation potential. Moreover, GBL isolates, particularly AI, showed antioxidant and anti-inflammatory activities in the use below the CC_50_ levels. Taken together, these results suggest that GBL isolates that are rich in antioxidant flavonoids are effective anti-neuroexocytotic agents with high stability and low toxicity.

## 1. Introduction

Flavonoids are known to have various health benefits, such as antioxidant, antimutagenic, antibacterial, antiangiogenic, anti-inflammatory, antiallergic, and anticancer activities [1,2,3]. Moreover, some flavonoids are previously reported to play a regulatory role in the release of neurotransmitters from neurons [4,5,6,7]. Flavonoids, including delphinidin, cyanidin, myricetin, and quercetin (Q), arrest the folding of the soluble *N*-ethylmaleimide-sensitive factor attachment protein receptor (SNARE) proteins [4,5,6,7]. The release of neurotransmitters from the neuronal cells can be regulated by the flavonoids, as SNARE complex formation is essential for the complete fusion between the synaptic vesicular membrane and plasma membrane. In vitro studies suggest that various stages of membrane fusion, including docking, hemifusion, and pore formation, are affected by the flavonoids because of direct interaction with both SNARE complex intermediate and membranes [4,7,8]. Flavonols, such as myricetin and Q, can mimic the activity of botulinum neurotoxins (BoNTs) by inhibiting SNARE complex formation since BoNTs block acetylcholine release by proteolyzing SNARE proteins [9]. Therefore, BoNTs are used in therapeutic interventions for diseases such as hyperhidrosis, wrinkles, joint and muscle pain, and other hypersecretion diseases.

*Ginkgo biloba* leaf (GBL) has been used as herbal remedies for centuries in East Asia. GBL is routinely used as one of the herbal nutraceuticals/supplements worldwide [10] and it is commonly used for improving ailments, including peripheral/cerebral circulation disorders, Alzheimer’s disease, short-term memory loss, depression, and anxiety [11]. Flavonoids are considered to be the primary bioactive components of the GBL extracts [12]. The occurrence of over 30 flavonoids, including flavonols, flavones, biflavones, and catechins, has been reported in the GBL [13]. To be used as functional foods, cosmetics, and pharmaceutical ingredients, flavonoid stability should be guaranteed, since the pH of the matrices/product formulations varies and heat is generated during production. Reportedly, glycosylation of flavonoids positively influences the physicochemical stability determined by the factors, such as the pH and thermal conditions [14]. It is also necessary to test the biocompatibility of the flavonoids before usage. Moreover, the functionalities, such as antioxidant and anti-inflammatory effects, can be additional merits of the GBL extracts.

Therefore, in this study, the pH and thermal stability of the flavonoids isolated from the GBL extracts were assessed, the neurotransmitter release inhibitory activity and toxicity were determined in *in vitro* and *in ovo* models, and the antioxidant and anti-inflammatory activities were also evaluated.

## 2. Results and Discussion

### 2.1. Identification of GBL Isolates

**Figure 1:**
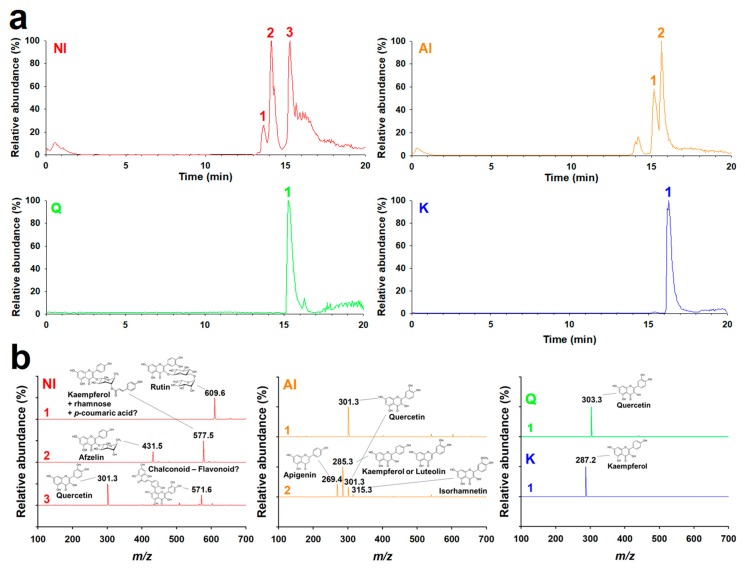
MS/MS analysis of the non-hydrolyzed isolate (NI) and acidolyzed isolate (AI) of *Ginkgo biloba* leaf. (**a**) Chromatograms and (**b**) mass spectra of NI ([M]^−^) and AI ([M]^−^), quercetin (Q; [M]^+^), and kaempferol (K; [M]^+^), following electrospray ionization in the HPLC-MS/MS measurements.

Flavonoids in the GBL extracts were isolated using acetone with or without H_2_SO_4_ solution and then purified by sedimentation. Figure 1 shows the purified isolates were identified with the high-performance liquid chromatography (HPLC)-MS/MS and ^1^H-NMR spectra. The HPLC-MS/MS chromatograms and mass spectra of the non-hydrolyzed isolate (NI), acidolyzed isolate (AI), and standard Q and kaempferol (K). The major peaks in the chromatograms of Q and K were observed at 15.3 and 16.3 min, respectively. Positively ionized mass spectra for these main peaks showed [M]^+^ (*m*/*z*) of 303.3 and 287.2 for Q and K, respectively. The data correspond well to the molecular weights (MWs) of Q (302.3) and K (286.2). Three major peaks were observed at 13.6, 14.1, and 15.3 min in the chromatogram of the NI. In the negatively ionized mass spectra, the peaks 1–3 exhibited [M]^−^ (*m*/*z*) of 609.6 for peak 1, 431.5 and 577.5 for peak 2, and 301.3 and 571.6 for peak 3. The calculated MWs corresponded well to the compounds previously identified in the GBL extracts. The MWs of 610.6, 432.5, 578.5, and 302.3 correspond to rutin (rutinoside of Q) [15], afzelin (rhamnoside of K) [16], unknown K glycosides (K + 2 rhamnose, K + 1 rhamnose + 1 *p*-coumaric acid, or K + 2 *p*-coumaric acid), and Q, respectively. The MW of 572.6 is discussed in the ^1^H-NMR spectra (Tables S1 and S2 and Figures S1 and S2). The above result suggests that the main compounds that are present in the NI are Q glycosides, K glycosides, Q aglycone, and others. The log *p* value of the NI was 0.76 ± 0.03, which was smaller than those of Q and K (2.59 ± 0.06 and 3.05 ± 0.13, respectively), owing to glycosides.

One minor and two major peaks were observed at 14.2, 15.2, and 15.6 min in the chromatogram of the AI. The negatively ionized mass spectrum indicated that the minor peak corresponded to K glycosides. Peak 1 of the AI, with an HPLC retention time of 15.2 min and [M]^−^ (*m*/*z*) of 301.3, was found to be Q. Peak 2 with a retention time of 15.6 min exhibited four [M]^−^ (*m*/*z*) of 269.4, 285.3, 301.3, and 315.3, which correspond to apigenin, K or luteolin, Q, and isorhamnetin, respectively. In contrast to the NI, the majority of the flavonoids in AI were aglycones. Accordingly, the log *P* value of the AI was determined to be 1.45 ± 0.06, which is almost twice greater than that of the NI. These differences clearly were due to the solvent used for the isolation of the AI, which contained H_2_SO_4_ for the removal of sugar moieties. To summarize, Q and K were abundant in both NI and AI, whereas a little of apigenin, luteolin, and isorhamnetin were found in AI. While most of the flavonoids in the NI were present as glycosides, including *p*-coumaroyl glycosides, the majority and minority of the flavonoids in the AI were aglycones and glycosides, respectively, owing to acidolyzing isolation.

### 2.2. Thermal and pH Stabilities of GBL Isolates

**Figure 2:**
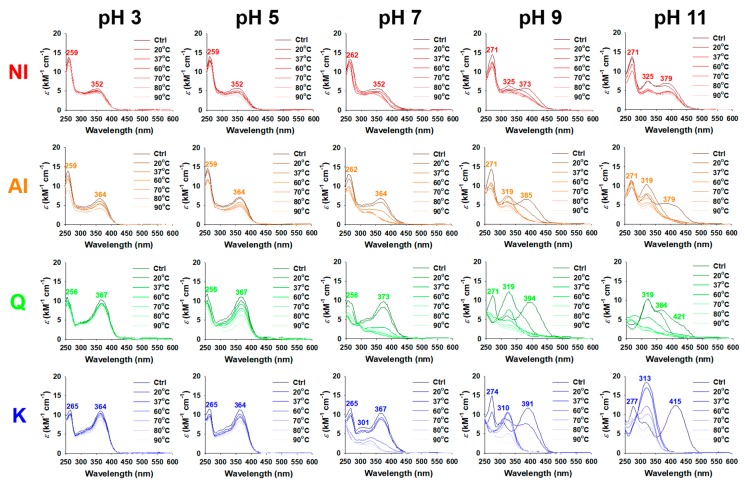
UV/visible spectra (250–600 nm) of the non-hydrolyzed isolate (NI) and acidolyzed isolate (AI) of *Ginkgo biloba* leaf, quercetin (Q), and kaempferol (K) at various pH ranges (3–11) and temperatures (20–90 °C) during 18-h incubation. Ctrl, control.

Flavonoids in the aqueous solution are sensitive to the environmental conditions, particularly temperature and pH. The changes in the UV/visible spectra of NI and AI were recorded under aqueous incubation conditions (20–90 °C and pH 3–11) for 18 h and compared with those of Q and K, abundant aglycones in NI and AI (Figure 2). At the initiation point of incubation, the absorption maxima (*λ*_max_) in the spectra of NI, AI, Q, and K were observed at different wavelengths. pH 3: 259/352 (NI), 259/364 (AI), 256/367 (Q), 265/364 nm (K); pH 5: 259/352 (NI), 259/364 (AI), 256/367 (Q), 265/364 nm (K); pH 7: 262/352 (NI), 262/364 (AI), 256/373 (Q), 265/301/367 nm (K); pH 9: 271/325/373 (NI), 271/319/385 (AI), 271/319/394 (Q), 274/310/367 nm (K); and pH 11: 271/325/379 (NI), 271/319/379 (AI), 319/364/421 (Q), 277/313/415 nm (K). As the pH increased, a red shift of the *λ*_max_ occurred (Figure 2). Similar to the results in this study, Q and its glycoside rutin had a red shift of the *λ*_max_ from the acidic to alkaline conditions [17].

Under stable conditions, the *λ*_max_ in the spectra of flavonoids that ranged 210–290 and 300–400 nm are typically associated with the light absorptive bands for the A-ring and B- and C-rings, respectively [18]. The *λ*_max_ 367–373 nm for Q and *λ*_max_ 364–367 nm for K at pH 3–7 were associated with the bands for the B- and C-rings. Furthermore, the *λ*_max_ 352 nm for NI and *λ*_max_ 364 nm for AI at pH 3–7 seem to arise from the B- and C-rings of aglycones (Q and K) and their glycosides. Rutin, a glycosylated form of Q, showed the *λ*_max_ 351 nm, a shorter wavelength when compared to that for Q (*λ*_max_ 367 nm) at pH 5 [17]. Furthermore, the maximum absorbance peak of NI at 352 nm indicates the abundance of the glycosylated forms of Q and K, in contrast to the maximum absorbance peak (364 nm) of the AI. In the spectra of NI and AI, the relatively large absorptivity intensity at < 290 nm and broad distribution of peaks at > 300 nm can be attributed to the various *λ*_max_ by some phenolic compounds, such as *p*-coumaric acid (285–333 nm) [19], apigenin (~270 and ~340 nm), luteolin (~250 and ~350 nm) [20], and isorhamnetin (255 and 368 nm) [21].

**Figure 3:**
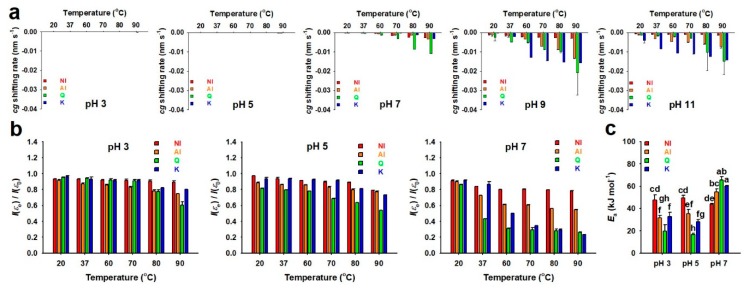
Stability of the non-hydrolyzed isolate (NI) and acidolyzed isolate (AI) of *Ginkgo biloba* leaf, quercetin (Q), and kaempferol (K) at various pH ranges (3–11) and temperatures (20–90 °C) during 18-h incubation. (**a**) Shifting rates of the center of gravity ($cg=\sum \varepsilon_{i}\lambda_{i}/\sum \varepsilon_{i}$; $\varepsilon_{i}$, absorptivity at i; $\lambda_{i}$, wavelength at i; 300–400 nm) in the UV-visible spectra of NI, AI, Q, and K. The cg was determined using the two segment linear fitting curves [$cg=k_{1}t+cg_{1}\left(0\leq t<t_{1}\right)=k_{2}t+cg_{2}\;\left(t_{1}\leq t\right)$] (Figure S7). Significantly different levels of the data sets are presented in Table S3. (**b**) Non-oxidized remaining ratios of integration values for the UV-visible spectra of NI, AI, Q, and K between the initiation ($I\left(\varepsilon_{0}\right)$) and end ($I\left(\varepsilon_{e}\right)$) of the incubations; the values for $I\left(\varepsilon_{e}\right)$ were determined using the exponential decay-plateau fitting curves [$\ln(I\left(\varepsilon \right)/I\left(\varepsilon_{0}\right))=-kt\;\left(0\leq t<t_{p}\right)=p\;\left(t_{p}\leq t\right)$] (Figure S8). Significantly different levels of the data sets are presented in Table S4. (**c**) Activation energy values ($E_{a}$) for NI, AI, Q, and K in the aqueous solutions at pH 3–7 and 20–90 °C. Data with different letters above the bars represent significant differences according to the Tukey’s test (*n* = 3; mean ± SD; *P* < 0.05).

In the alkaline condition, the deprotonation of phenolic hydroxyl groups in Q and K initiates the formation of quinone intermediates, which allows for them to be easily autoxidized [22]. We observed, immediately after incubating under alkaline conditions (pH 9 and 11), some *λ*_max_ of Q and K near the violet- and blue-light regions (380–485 nm), indicating that the autoxidation of Q and K occurred, unlike that under the acidic and neutral conditions. After incubation under the alkaline conditions for 18 h, as temperature increased from 20 to 90 °C, the absorbance of Q and K at the long wavelengths (380–485 nm) decreased, while the peaks at the wavelengths that ranged 310–319 nm first increased for a specific temperature, and then decreased (Figure 2). The observed maximum absorbance peaks at 310–319 nm were associated with the formation and destruction of the first-stage oxidative products, such as 2-(3,4-dihydroxybenzoyl)-2,4,6-trihydroxybenzofuran-3-one and 2,3-dihydroxyflavanone [22]. The first-stage oxidative products are further oxidized to the second-stage oxidative products, such as 2,4,6-trihydroxybenzoic acid, protocatechuic acid, and 4-hydroxybenzoic acid [22]. The absorption bands derived from the first- and second-stage oxidative products were also observed in the spectra of Q and K after incubation in pH 7, at >60 °C (Figure 2). In contrast, no absorption band for oxidative products of the first- and second-stages was observed in all of the spectra for incubations at pH 3 and 5, while the absorbance decreased in the entire wavelength range of the spectra. Similar autoxidation phenomena were observed in the spectra of NI and AI under all of the incubation conditions in this study, except for slight differences in the wavelength and extent of the maximum absorbance peaks as compared to those of Q and K. The band shifts and kinetics were determined (Figure 3) based on time-dependent changes in the UV-visible spectra of NI, AI, Q, and K to study these complicated oxidation phenomena (Figures S3–S6).

The band shifting rates in the spectra of NI, AI, Q, and K were determined through regression fitting with the cg values (Figure S7). All of the cg shifting rates had negative values, indicating blue shifts of bands in all the spectra for NI, AI, Q, and K (Figure 3a). All the shifting rates in the acidic and neutral conditions were close to zero, except those of Q at > 80 °C, although, in the alkaline condition, the blue shifting rates increased with elevation of the incubation temperature, regardless of samples. Particularly, the blue shifting rates of AI, Q, and K incubated at pH 9 and 11 were higher than those at the other pH conditions, regardless of temperature, which indicated their unstable property in alkaline aqueous solutions. On the other hand, under most of the incubation conditions, the blue shifting rates of NI were the lowest among all the samples, suggesting that NI is more stable as compared to AI, Q, and K. This might be attributed to the abundance of glycosylated flavonoid forms in NI, unlike the others [17].

In addition to the band shifting rate, the non-oxidized remaining ratios of NI, AI, Q, and K were determined through regression fitting with the integration values of the spectral bands to verify the effects of the incubation conditions on oxidation (Figure S8). Since complete autoxidation occurred in an instant in the alkaline incubation conditions, the non-oxidized remaining ratios were only determined in the acidic and neutral conditions (Figure 3b). In all of the samples used in this study, the remaining ratios decreased as the temperature increased. Particularly, the remaining ratios of NI were the highest in most of the incubation conditions, and ~78% of NI remained, even after incubating at pH 7 and 90 °C, whereas ~26% of Q and ~23% of K remained. In all of the conditions, the remaining ratios of AI were lower than those of NI, presumably because of the effect of deglycosylation by acidolysis. Based on the determination of $E_{a}$ values (Figure S9), the $E_{a}$ values of NI were maintained at 44–48 kJ·mol^−1^, regardless of pH (Figure 3c). However, the $E_{a}$ values of AI significantly increased as the pH increased from 5 to 7 (~35 to ~55 kJ·mol^−1^), which was also observed in Q and K (Figure 3c). The results above suggest the NI has higher stability in aqueous conditions, regardless of pH and temperature when compared to the others (Figure 3), partly due to more abundant glycosylated flavonoid forms.

### 2.3. Neurotransmitter-Release Inhibitory Activity and Biocompatibility of GBL Isolates

**Figure 4:**
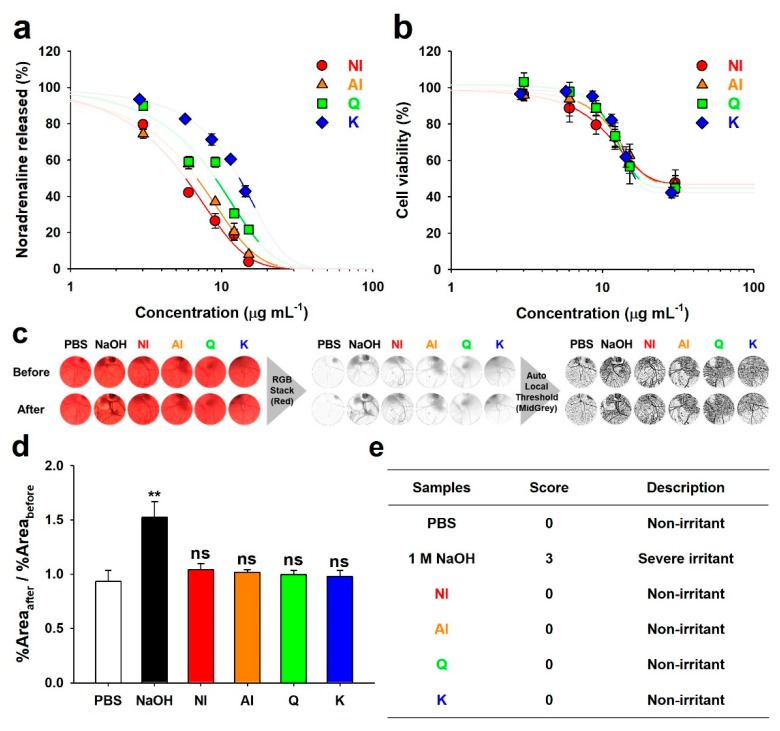
Neurotransmission inhibitory efficacy and safety of the non-hydrolyzed isolate (NI) and acidolyzed isolate (AI) of *Ginkgo biloba* leaf, quercetin (Q), and kaempferol (K). (**a**) Inhibition of norepinephrine release from the differentiated PC-12 cells by NI, AI, Q, and K. (**b**) Cytotoxicity of NI, AI, Q, and K towards the PC-12 cells. Thick lines on the fitting curves represent the region under both EC_50_ and CC_50_ values. (**c**) Images of the hen egg’s chorioallantoic membranes before and after (1 min) each treatment. Two milliliters of phosphate-buffered saline (PBS), 1 M NaOH solution (NaOH), and 1 mg·mL^−1^ solutions of NI, AI, Q, and K were used for the treatment. Image processing procedures to determine the area of blood vessels are shown. (**d**) Ratios of the black pixel area fraction (%Area) before and after sample treatments (Student’s *t*-test with the data of PBS: **, *P* < 0.01; ns, *P* > 0.05). (**e**) Scores obtained from the Hen’s egg test-chorioallantoic membrane (HET-CAM).

Some flavonoids, including myricetin, Q, fisetin, and luteolin, possess characteristics inhibiting neuronal SNARE-complex formation that induces the release of neurotransmitters to synaptic clefts [7]. Moreover, norepinephrine that was released from differentiated PC-12 cells, as simulated neurons, was quantified to verify the inhibitory effects of the neurotransmitter release by flavonoids in NI and AI (Figure 4a). From the curves in Figure 4a, the 50% effective concentrations (EC_50_) were calculated (Table 1). The EC_50_ of NI (5.75 μg·mL^−1^) was lower than those of AI, Q, and K (6.84, 9.05, and 13.11 μg·mL^−1^, respectively), which suggested greater neurotransmitter-release inhibitory activity of NI. A relatively higher EC_50_ value of K indicates lower norepinephrine release inhibitory activity, which is in agreement with the results of our previous study [7].

According to our previous results that were obtained with protein level assays, *in vitro* assays using the SNARE proteins’ complexation, Q significantly exhibited stronger inhibitory activity of the SNARE complex formation than K and the glycosides of Q, such as isoquercetrin, spiraeoside, and rutin [7]. The majorities of Q and K in NI are present as the glycosidic forms, as explained above (Figure 1 and Figure S1). Besides, minor compounds such as biflavonoids were negligibly present in NI. Accordingly, pure aglyconic Q should have exhibited greater inhibition efficacy to the neurotransmitter release than NI. However, in this study, the EC_50_ of NI was lower than that of Q, suggesting that the efficacy of NI was greater than that of pure aglyconic Q. This result is not corresponded with our previous results using the protein level assays [7]. Thus, this result can bring a hypothesis that other unknown effects, which might be attributed to differences in the assay, such as the use of live cells and the incubation with the samples, can influence this phenomenon. In this regard, the strong inhibitory activity of NI might be attributed to the great stability or active transport by neuronal glucose transporters [23]. Additionally, K is known to strongly inhibit β-hexosaminidase or histamine release from mast cells by inhibiting the signaling pathway, but not affecting the SNARE-driven fusion [24]. In present study, the weak norepinephrine release inhibitory activity of K might result from the inhibition of the signaling pathway in the differentiated PC-12 cells, not from the inhibition of the SNARE complexation. Consequently, the relatively lower EC_50_ values of NI and AI as compared to those of each Q and K can be attributed to the synergetic inhibition effects of aglyconic and glycosidic Q and K that are present in AI and NI on SNARE-driven fusion and an unknown signaling pathway, respectively.

The cytotoxic effects of NI and AI on the PC-12 cells were evaluated (Figure 4b) and the 50% cytotoxic concentrations (CC_50_) were calculated (Table 1). The CC_50_ values of NI and AI were 28.99 and 23.31 μg·mL^−1^, respectively, which were not significantly different from those of Q and K (17.75 and 16.61 μg·mL^−1^, respectively). The ratio of EC_50_ to CC_50_ (EC_50_/CC_50_) was determined as an indicator of the therapeutic window. The EC_50_/CC_50_ values of NI and AI were 0.26 and 0.30, respectively, which revealed a wider therapeutic window than those of Q and K (0.52 and 0.79, respectively). Therefore, NI and AI can be advantageous as nerve agonists. Moreover, in the Hen’s egg test-chorioallantoic membrane (HET-CAM) (Figure 4c–e), the *in ovo* ocular nontoxicity of all the samples was determined, which demonstrated the biocompatibility for their use as facial cosmetics or pharmaceutical ingredients.

### 2.4. Antioxidant and Anti-Inflammatory Activities of GBL Isolates

**Figure 5:**
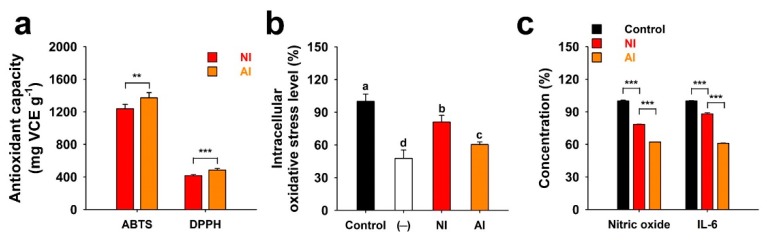
Antioxidant and anti-inflammatory activities of the non-hydrolyzed isolate (NI) and acidolyzed isolate (AI) of *Ginkgo biloba* leaf. (**a**) Antioxidant capacity expressed as vitamin C equivalents (VCE)·g^−1^ of NI and AI determined using ABTS and DPPH radicals. (**b**) H_2_O_2_ (100 μM)-induced oxidative stress levels of the PC-12 cells treated with 10 μg·mL^−1^ of NI and AI [Control, no sample-treated group; (−), no H_2_O_2_- or sample-treated group]. Data with different letters represent significant differences according to the Tukey’s test (*n* ≥ 3; mean ± SD; *P* < 0.05). (**c**) Lipopolysaccharide (100 ng·mL^−1^)-induced generations of nitric oxide and interleukin 6 (IL-6) from the RAW 264.7 macrophages treated with 20 μg·mL^−1^ of NI and AI. Student’s *t*-test: **, *P* < 0.01; ***, *P* < 0.001.

NI and AI showed 2,2′-azino-bis(3-ethylbenzothiazoline-6-sulfonic acid) (ABTS) radical scavenging activity of 1238.2 and 1372.1 mg vitamin C equivalents (VCE)·g^−1^, respectively, whereas they had 2,2-diphenyl-1-picrylhydrazyl (DPPH) radical scavenging activity of 416.2 and 484.8 mg VCE·g^−1^ (Figure 5a). The ABTS and DPPH radical scavenging activities of AI was higher than NI, due in part to the fact that higher amounts of flavonoid aglycones are present in AI as compared to its counterpart. It was previously reported that flavonoid glycosides show lower antioxidant activities than their aglycones [25]. The levels of intracellular oxidative stress were approximately 61% for AI and 77% for NI when compared to the control group (100%) (Figure 5b). Flavonoid aglycones, such as Q, can easily penetrate the cellular membrane in a simple diffusional way due to its high hydrophobic property than their glycosides [25,26]. Similar to the results of antioxidant activities in this study (Figure 5a), therefore, AI had a higher decrease of intracellular oxidative stress than NI. These results contrast to the result in the neurotransmission inhibitory assay. The greater efficacies of AI than those of NI can result from the higher antioxidant and intracellular oxidative stress-lowering activities of the flavonoid oxides or the use of non-differentiated PC-12.

Nitric oxide (NO) release of RAW 264.7 macrophages was measured based on the accumulation of nitrite, a stable metabolite in culture supernatants [27]. Although unstimulated macrophages produce undetectable levels of NO and interleukin 6 (IL-6), treatments of RAW 264.7 macrophages with lipopolysaccharide (LPS) increased the production of NO and IL-6 (Figure 5c). NI and AI both significantly lowered NO and IL-6 production as compared to groups that were only treated with LPS (Figure 5c). Like the results of antioxidant capacities and intracellular oxidative stress in this study (Figure 5a,b), AI rich in flavonoid aglycones had higher anti-inflammatory effects than flavonoid-glycosides-rich NI. Activated macrophages secrete several inflammatory mediators, including NO and IL-6 [27]. However, excessive and persistent production of these inflammatory mediators is known to be involved in several inflammatory diseases and cancer [28]. Therefore, reducing the NO and IL-6 generation by inhibiting the activation of macrophages is important in the treatment of inflammatory diseases. Previous studies have reported that flavonoid aglycones have more effective anti-inflammatory effects than flavonoid glycosides [29,30]. Flavonoid glycosides may have steric hindrance due to their bulky glycosyl residues that are attached to flavonoid backbone or may not easily penetrate the cell membrane due to their high hydrophilicity [31]. Antioxidative polyphenols are reported to effectively reduce inflammation due to their scavenging of reactive oxygen species [32]. Therefore, AI showing a high amount of antioxidative flavonoid aglycones may contribute to the reduction of inflammatory mediators-formation in RAW 264.7 macrophages.

**Figure 6:**
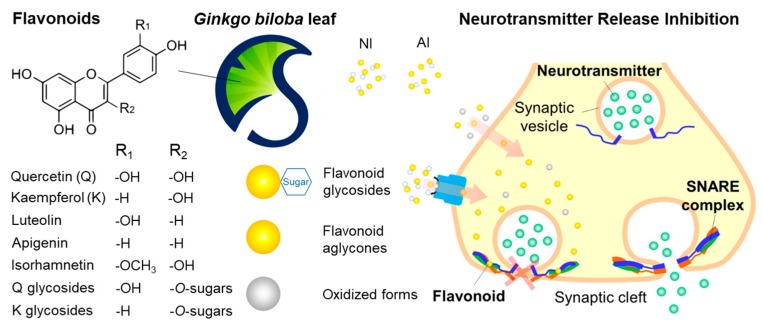
Graphical representation of the proposed mechanism on the neurotransmission inhibitory activities of the non-hydrolyzed isolate (NI) and acidolyzed isolate (AI) of *Ginkgo biloba* leaf.

To summarize, GBL NI and AI, particularly NI, have the significantly greater neurotransmission inhibitory activities compared to Q and K, and both NI and AI were low toxic. With respect to the neurotransmission inhibition, NI is more efficient than AI due to the higher pH-/thermo-stability or possible active transport through the cell membrane, which is contributed to the richer flavonoid glycosides (Figure 6). In terms of the antioxidant, intracellular oxidative stress-lessening, and anti-inflammatory activities, AI is more effective than NI, because the oxidized flavonoid have greater activities or the richer aglycones make more diffusible through the cell membrane.

## 3. Materials and Methods

### 3.1. Chemicals

GBL powder (batch number: GB0170920) was purchased from TnV International Inc. (Chino, CA, USA), which contained 28.52% of total flavonoid content, 6.02% of total lactone content, and 6.63% of the sum of ginkgolide A to C and bilobalide according to the information that was supplied by the vendor. Q, K, ABTS, DPPH, 2′,7′-dichlorofluorescin diacetate (DCFH-DA), and neutral red were obtained from Sigma Aldrich Co., LLC (St. Louis, MO, USA). Acetone and dimethyl sulfoxide (DMSO) were purchased from Dae Jung Chemicals (Siheung, Korea). Phosphate-buffered saline (PBS), 1 vol% penicillin-streptomycin and Hanks’ balanced salt solution (HBSS) were obtained from Welgene Inc. (Gyeongsan, Korea). Dulbecco’s modified eagle medium (DMEM) and fetal bovine serum (FBS) were obtained from GE Healthcare (Chicago, IL, USA). Nerve growth factor (NGF mouse protein 7S subunit) and 1 vol% antibiotic-antimycotic (10×) solution were purchased from GIBCO/Invitrogen (Grand Island, NY, USA). All other chemicals were of analytical and reagent grade.

### 3.2. Isolation and Purification of Flavonoids from GBL Extracts

Flavonoids were isolated from the GBL powder while using two different methods. In the first method, 4 g of the powder was incubated with 40 mL acetone at 50 °C for 3 h, passed through a filter paper, and then powderized by acetone-evaporation in a rotavapor (R-100; BÜCHI, Rheinstetten, Germany). In the second method, 4 g of the powder was incubated with 40 mL acetone and 100 μL of 0.1 M H_2_SO_4_ aqueous solution at 50 °C for 3 h, passed through a filter paper, and then concentrated by acetone-evaporation in the rotavapor (R-100; BÜCHI).

The concentrate was dissolved in 80 mL double-deionized water (DDW) and centrifuged (10 min, 25 °C, 6000 relative centrifugal force), and the sediment was collected after removing the supernatant to remove the hydrophilic compounds (lactone, ginkgolide, bilobalide, sugars, etc.) except the flavonoids. This dissolution/sedimentation procedure was repeated twice with the collected sediment, and the final sediment was obtained, which was powderized in the rotavapor and then stored in −80 °C before further experiments. The isolates that were obtained by the first and second isolation methods were named as NI and AI, respectively.

### 3.3. HPLC-MS/MS

HPLC analyses were conducted with an Ultimate 3000 RS module system (Thermo Fisher Scientific Inc., Waltham, MA, USA) connected to an LTQ-orbitrap classic (Thermo Fisher Scientific Inc., Waltham, MA, USA). The chromatograms and mass spectra were obtained to identify NI and AI (Figure 1). Briefly, 2 μL sample solution was separated in a reversed-phase column (U-VD Spher Pur C18-E, 1.8 μm, 100 × 2.0 mm; VDS Optilab, Berlin, Germany) at 40 °C for 20 min with a gradient setting of the mobile phase (Table S5). The effluent from the HPLC was directly transferred into the mass spectrometer through electrospray-ionization (capillary temperature, 300 °C; source voltage, 3.5 kV). The survey full-scan MS spectra (*m/z*, 100–2000) were acquired in the orbitrap.

### 3.4. Assessment of Thermal and pH Stability

UV/visible spectra of NI and AI at the wavelength range of 250–600 nm were obtained using a microplate reader to assess the thermal and pH stabilities in an aqueous environment (measuring interval of 3 nm, 25 °C; Synergy H1; BioTek Instruments Inc., Winooski, VT, USA). Hundred microliters of NI and AI (30 μg·mL^−1^), dissolved in DMSO, were mixed in a micro-tube (2 mL) with 1.9 mL PBS, pre-adjusted to pH 3–11 with 1 M HCl or NaOH aqueous solution. The mixture was incubated in a heating drybath (S08040; Thermo Scientific^TM^, Marietta, OH, USA) at pre-determined temperatures (20–90 °C) with shaking at 700 rpm. At the pre-determined time points (0–18 h), 200 μL incubated mixtures were transferred into the wells of a 96-well microplate, and their light absorptive spectra were then recorded while using Synergy H1 (BioTek Instruments Inc.). Q and K of the same concentration in NI and AI were also used to compare the stability.

The spectrum that was composed of each absorptivity (*ε*, M^−1^·cm^−1^) value at a specific wavelength was correlated with the non-oxidized amounts of NI, AI, Q, and K in the incubated mixture following the Beer–Lambert law. The non-oxidized amounts of NI, AI, Q, and K cannot be only calculated from the absorptivity at a specific wavelength since the spectrum patterns of NI, AI, Q, and K varied with the pH conditions (Figure 2). Therefore, the non-oxidized amount was determined based on the integration values [I(ε)] of the absorptivity in a wavelength range of 250–600 nm at the incubation time (t) instead of the absorptivity values at a specific wavelength. An exponential decay-plateau fitting method was applied in order to determine the decay rate constant values (k, s^−1^) for NI, AI, Q, and K, incubated at various circumstances, as follows:

(1) $$\ln\left[I\left(\varepsilon \right)/I\left(\varepsilon_{0}\right)\right]=-kt\;\left(0\leq t<t_{p}\right)=p\;\left(t_{p}\leq t\right)$$

where $I\left(\varepsilon_{0}\right)$ is the integration value of the absorptivity at the initiation of incubation and $t_{p}$ is the time when the left side (−kt) is about to be plateau constant (p). The non-oxidized remaining ratios of NI, AI, Q, and K after incubations were described as $e^{p}$. Based on the k values, activation energy ($E_{a}$) for decay of NI, AI, Q, and K in aqueous solution at pH 3–11 was determined while using the Arrhenius equation, as follows:

(2) $$\ln k=-\left(E_{a}/R\right)\left(1/T\right)+\ln A$$

where R is the ideal gas constant (8.3144598 J·mol^−1^·K^−1^), T is the temperature (K), and A is the pre-exponential constant.

Monitoring the shift of specific peaks in the spectra for NI, AI, Q, and K is important for applications in foods and cosmetics as the shift of specific peaks indicates color changes, and customers usually do not prefer unwanted color changes of the products. Therefore, the center of gravity in the spectra for NI, AI, Q, and K was determined in order to monitor the shift of specific peaks: $\mathrm{center}\;\mathrm{of}\;\mathrm{gravity}\;\left(cg\right)=\sum \left(\varepsilon_{i}\lambda_{i}\right)/\sum \varepsilon_{i}$, where $\varepsilon_{i}$ is the absorptivity at a specific wavelength ($\lambda_{i}$, 300–400 nm). Consequently, the shifting rate of the cg was estimated during the incubation of NI, AI, Q, and K.

### 3.5. Cell Cultivation

Neuronal PC-12 cells, which were obtained from Korean Cell Line Bank (Seoul, Korea), were cultured in DMEM, being supplemented with 10 vol% heat-inactivated FBS, and 1 vol% antibiotic-antimycotic (10×) solution. Murine macrophage RAW 264.7 cells, which were obtained from American Type Culture Collection (Manassas, VA, USA), were cultured in DMEM, supplemented with 10 vol% heat-inactivated FBS and 1 vol% penicillin-streptomycin. The cells were incubated at 37 °C under humidified air composition of 5% CO_2_. The culture media were replaced every other day. Prior to experiments, the Trypan Blue dye exclusion test with a manual hemocytometer was utilized to assess the viability of the PC-12 and RAW 264.7 cells in 1–5 and 10–15 passages, respectively.

### 3.6. Norepinephrine Release Assay

The harvested PC-12 cells were seeded onto 24-well plates that were coated with poly-d-lysine at a density of 2 × 10^5^ cells per well and grown in the culture media for five days. Subsequently, the cells were treated with the nerve growth factor (50 ng·mL^−1^) and incubated for seven days. The media was removed, and 1 mL high-K^+^ media (115 mM NaCl, 50 mM KCl, 1.2 mM KH_2_PO_4_, 2.5 mM CaCl_2_, 1.2 mM MgSO_4_, 11 mM glucose, and 15 mM Hepes-Tris; pH 7.4) was added to the wells, and then washed with the low-K^+^ media (140 mM NaCl, 4.7 mM KCl, 1.2 mM KH_2_PO_4_, 2.5 mM CaCl_2_, 1.2 mM MgSO_4_, 11 mM glucose, and 15 mM Hepes-Tris; pH 7.4) 15 min later. Subsequently, 1 mL of 1 vol% DMSO in the low-K^+^ media, containing the NI, AI, Q, and K, was added to the well and then incubated for 4 h. Next, the cells were washed to remove the extracellular compounds along with the low-K^+^ media and treated again with the high-K^+^ solution to depolarize the cells and stimulate neurotransmitter release. After 15 min, norepinephrine released from the cells into the media was quantified using the norepinephrine enzyme-linked immunosorbent assay (ELISA) kits (IBL International, Hamburg, Germany) and a microplate reader (*λ* = 405 nm; Synergy H1; BioTek Instruments Inc.). The amount of the released norepinephrine was determined by subtracting the basal level signal from the signal of the sample-treated cells.

### 3.7. Determination of Cell Viability

The harvested PC-12 cells (1.0 × 10^4^ cells per well) were incubated in the culture media to adhere to the 96-well plates for five days. After incubation, the PC-12 cells were washed using fresh DMEM, added with 200 μL DMEM, and then incubated for 12 h. The medium was removed, and 100 μL of 0.33 wt% neutral red solution (dissolved in DMEM) were added into the wells and incubated for 2 h. The solution was carefully removed, and the cells were rinsed with PBS (pH 7.4) and then completely dried at the ambient condition. Next, the neutral red was dissolved by adding 200 μL solubilizing solution (50 vol% ethanol, 49 vol% DDW, and 1 vol% acetic acid) and shaken, and the absorbance at 540 nm was then measured using the Synergy H1 microplate reader (BioTek Instruments Inc.).

### 3.8. HET-CAM

HET-CAM has not been officially accepted yet as an Organization for Economic Co-operation and Development Test Guideline. However, this test is generally recognized as a scientifically reasonable testing method for screening ophthalmic irritancy [33] and has been validated by an European Union Directive on dangerous substances [34]. In this study, all HET-CAM experiments, slightly modified based on a previous study [34], were performed according to a protocol that was reported from an Interagency Coordinating Committee on the Validation of Alternative Methods of the National Institutes of Health [35]. Fertilized hen eggs were tested in accordance with the approval and guidelines of the Institutional Animal Care and Use Committee of Sungkyunkwan University (SKKUIACUC2018-04-11-2). All of the experimental protocols were approved by animal care and use committee of Sungkyunkwan University. For the HET-CAM, fertilized hen eggs were obtained from a local market in Korea and then incubated for 10 days (37.5 °C and 45% humidity), maintaining auto-rotation at 90°·h^−1^ to ensure the proper development and viability of the embryo. After incubation, the upper shell of the embryo-formed eggs was cut in a circular shape, avoiding damage to the inner membrane. The inner membrane was carefully removed with forceps to avoid injury to the blood vessels, and the CAM was then treated with 2 mL test-sample solutions (AI, NI, K, and Q). Prior to the treatment, the test-sample solutions were prepared by mixing 1.9 mL PBS and 100 μL DMSO containing NI, AI, Q, and K, to prevent the sedimentation of the crystalline solute. The mixture of PBS (1.9 mL) with DMSO (100 μL) and 1 M NaOH aqueous solution were used as the positive and negative controls, respectively. Next, using a flashlight on the bottom of the eggshell, the CAM images were obtained after 1 min from the addition of sample-solutions.

Based on the CAM images, the severity of any hemorrhage, blood-coagulation, and hyperemia was graded from 0 (no irritation) to 3 (strong irritation) while using a previously developed scoring method [34]. We analyzed the CAM images using ImageJ (available at http://rsb.info.nih.gov/ij/) to prevent arbitrary judgments on the previously developed scoring method, selected the blood vessels in the CAM images, and measured hemorrhage, blood-coagulation, and hyperemia, as follows. The CAM images were individually loaded onto ImageJ, cropped to a fixed size to exclude the shell, and converted into greyscale. The greyscale CAM images on the red stack were adjusted using the MidGrey method, and the area fraction (%area) values of the black pixels in the adjusted CAM images were measured and recorded.

### 3.9. Determination of Antioxidant Capacity and Anti-Inflammatory Effect

Antioxidant capacity was measured using the ABTS and DPPH radicals [36]. The antioxidant capacity was expressed as mg VCE·g^−1^ of dried sample. In the ABTS radical scavenging assay, the ABTS radical solution was adjusted to an absorbance of 0.650 ± 0.020 at 734 nm. The reaction between ABTS radicals and samples was allowed to set at 37 °C for 10 min, and the decrease in absorbance of the resulting solution was then measured at 734 nm while using a SPECTRONIC 200 spectrophotometer (Thermo Fisher Scientific Inc., Waltham, MA, USA). In the DPPH radical scavenging assay, the DPPH radicals (0.1 mM) were dissolved in 80 vol% aqueous methanol. The absorbance of DPPH radicals was set to 0.650 ± 0.020 at 517 nm. The reaction between DPPH radicals and samples was allowed to proceed at 23 °C for 30 min. The decrease in absorbance of the resulting solution was monitored at 517 nm while using a SPECTRONIC 200 spectrophotometer (Thermo Fisher Scientific Inc.).

The level of intracellular oxidative stress was evaluated using the fluorescent probe DCFH-DA [37]. PC-12 cells (2 × 10^4^ cells per well) were pre-cultured for 24 h and then treated with NI and AI (10 μg·mL^−1^) for 24 h. After removing the supernatant, the PC-12 cells were incubated with 50 μM DCFH-DA in HBSS for 1 h. The PC-12 cells were treated with 100 μM H_2_O_2_ in HBSS for 1 h. Fluorescence was measured at 485 nm (detection wavelength) and 535 nm (emission wavelength) using a microplate reader (Infinite M200; Tecan Austria GmbH, Grödig, Austria). The intracellular oxidative stress level was expressed as the percentage (%) of decrease in fluorescence intensity of control (100%).

The production of NO and IL-6 was measured to confirm the anti-inflammatory effects of samples [27,38]. For measurements of NO production, RAW 264.7 cells at a density of 1 × 10^5^ cells per a well in 48-well plate were pre-cultured for 24 h. The cells were stimulated with 100 ng·mL^−1^ LPS in the presence of NI or AI (20 μg·mL^−1^) for 24 h. The supernatant was obtained for the evaluation of nitrite level (an indicator of NO production) by using the Griess reagent system and measuring the absorbance at 540 nm with the Infinite M200 (Tecan Austria GmbH). For quantifying IL-6 production, the RAW 264.7 cells at a density of 4 × 10^5^ cells per a well in 24-well plate were pre-cultured for 24 h. The cells were stimulated with 100 ng·mL^−1^ LPS in the presence of NI or AI (20 μg·mL^−1^) for 24 h. IL-6 levels of the supernatant were determined using the ELISA kits according to the manufacturer’s protocol (BD Biosciences, San Jose, CA, USA). NI and AI did not affect the viability of RAW 264.7 cells up to 20 μg·mL^−1^ (data not shown), which indicated that their inhibitory effects of the production of NO and IL-6 are not due to their cytotoxic effects.

### 3.10. Statistical Analyses

All of the data represent an average of at least three independent experiments or measurements. Data are presented as mean ± standard deviation. The kinetic parameters and the curves were estimated and fitted using a linear or nonlinear regression iteration procedure using SigmaPlot (V10.0; IBM Co., Armonk, NY, USA). Statistical analyses were firstly started by examination of the data using the distribution normality test (Shapiro–Wilk test) and the variance homogeneity test (Levene’s test). If normal distribution and homogeneous variance were guaranteed, further statistical analyses (Tukey’s test or Student’s *t*-test) were conducted using SPSS Statistics (V23.0; IBM Co., Armonk, NY, USA).

## 4. Conclusions

The flavonoids abundantly present in NI were glycosides of Q and K, whereas the primary flavonoids in AI were aglyconic Q and K. The presence of flavonoid glycosides in NI and AI derived from GBL, particularly NI, imparted pH and thermal stabilities in aqueous conditions. NI and AI had a higher inhibitory activity of neurotransmitter release than flavonol aglycones (Q and K). The analysis of HET-CAM results revealed that both NI and AI were biocompatible, which suggested their applicability as ingredients for facial treatment. Moreover, GBL isolates, particularly AI, showed antioxidant capacity and anti-inflammatory effects below the CC50 levels, which is additional merit for utilizing as food, cosmetic, and pharmaceutical ingredients. Collectively, GBL isolates (NI and AI) may serve as functional materials to develop stable, effective, safe, and multifunctional ingredients for improving the negative symptoms that are induced by excess neurotransmissions, such as hyperhidrosis, wrinkles, and pain.

## Figures and Tables

**Table 1:** EC_50_, CC_50_, and EC_50_/CC_50_ values of the non-hydrolyzed isolate (NI) and acidolyzed isolate (AI) of *Ginkgo biloba* leaf, quercetin, and kaempferol upon treatment of the differentiated PC-12 cells. ^1.^

Samples	EC_50_ (μg·mL^−1^)	CC_50_ (μg·mL^−1^)	EC_50_/CC_50_
NI	5.75 ± 0.10 ^a^	28.99 ± 6.07 ^a^	0.26 ± 0.07 ^a^
AI	6.84 ± 0.38 ^b^	23.31 ± 5.14 ^a^	0.30 ± 0.07 ^b^
Quercetin	9.05 ± 0.18 ^c^	17.75 ± 3.59 ^a^	0.52 ± 0.09 ^c^
Kaempferol	13.11 ± 0.45 ^d^	16.61 ± 1.09 ^a^	0.79 ± 0.03 ^d^

^1^ EC_50_: 50% effective concentration; CC_50_: 50% cytotoxic concentration; data with different letters in the same column represent significant differences according to the Tukey’s test (*n* = 3; mean ± SD; *P* < 0.05).

## Supplementary Materials

The following are available online, Figure S1: ^1^H-NMR spectra of the *Ginkgo biloba* leaf (GBL) isolates; Tables S1 and S2: the expected ^1^H chemical shifts (δ) of the proposed chemical structures of flavonoid-flavonoid, chalconoid-flavonoid, flavonoid-chalconoid, and coumaric acid; Figure S2: proposed chemical structures of flavonoid-flavonoid, chalconoid-flavonoid, flavonoid-chalconoid, and coumaric acid; Figures S3–S6: UV/visible spectra of the GBL isolates; Figures S7 and S8: changes in the cg and absorptivity in the UV/visible spectra of the GBL isolates; Figure S9: decay kinetics of the GBL isolates; Tables S3 and S4: significantly different levels of the *cg* shifting rate and non-oxidized remaining amount [$I\left(\varepsilon \right)/I\left(\varepsilon_{0}\right)$] in the UV/visible spectra of the GBL isolates; Table S5: mobile phase composition for the HPLC-MS/MS.

## References

[1] García-Lafuente A., Guillamón E., Villares A., Rostagno M.A., Martínez J.A. (2009). Flavonoids as anti-inflammatory agents: implications in cancer and cardiovascular disease. Inflamm. Res..

[2] Ravishankar D., Rajora A.K., Greco F., Osborn H.M.I. (2013). Flavonoids as prospective compounds for anti-cancer therapy. Int. J. Biochem. Cell Biol..

[3] Cushnie T.P.T., Lamb A.J. (2011). Recent advances in understanding the antibacterial properties of flavonoids. Int. J. Antimicrob. Agents.

[4] Heo P., Park J.-B., Shin Y.-K., Kweon D.-H. (2017). Visualization of SNARE-mediated hemifusion between giant unilamellar vesicles arrested by myricetin. Front. Mol. Neurosci..

[5] Heo P., Yang Y., Han K.Y., Kong B., Shin J.-H., Jung Y., Jeong C., Shin J., Shin Y.-K., Ha T. (2016). A chemical controller of SNARE-driven membrane fusion that primes vesicles for Ca2+-triggered millisecond exocytosis. J. Am. Chem. Soc..

[6] Yang Y., Choi J.K., Jung C.H., Koh H.J., Heo P., Shin J.Y., Kim S., Park W.-S., Shin H.-J., Kweon D.-H. (2012). SNARE-wedging polyphenols as small molecular botox. Planta Med..

[7] Yang Y., Shin J.Y., Oh J.-M., Jung C.H., Hwang Y., Kim S., Kim J.-S., Yoon K.-J., Ryu J.-Y., Shin J. (2010). Dissection of SNARE-driven membrane fusion and neuroexocytosis by wedging small hydrophobic molecules into the SNARE zipper. Proc. Natl. Acad. Sci. USA.

[8] Yang Y., Heo P., Kong B., Park J.-B., Jung Y., Shin J., Jeong C., Kweon D.-H. (2015). Dynamic light scattering analysis of SNARE-driven membrane fusion and the effects of SNARE-binding flavonoids. Biochem. Biophys. Res. Commun..

[9] Pirazzini M., Rossetto O., Eleopra R., Montecucco C. (2017). Botulinum neurotoxins: biology, pharmacology, and toxicology. Pharmacol. Rev..

[10] Mahadevan S., Park Y. (2008). Multifaceted therapeutic benefits of *Ginkgo biloba* L.: chemistry, efficacy, safety, and uses. J. Food Sci..

[11] Kleijnen J., Knipschild P. (1992). *Ginkgo biloba* for cerebral insufficiency. Br. J. Clin. Pharmacol..

[12] Kleijnen J., Knipschild P. (1992). Ginkgo biloba. Lancet.

[13] Zhang Q., Chen L.J., Ye H.Y., Gao L., Hou W., Tang M., Yang G., Zhong Z., Yuan Y., Peng A. (2007). Isolation and purification of ginkgo flavonol glycosides from *Ginkgo biloba* leaves by high-speed counter-current chromatography. J. Sep. Sci..

[14] Hostetler G.L., Riedl K.M., Schwartz S.J. (2013). Effects of food formulation and thermal processing on flavones in celery and chamomile. Food Chem..

[15] Beck S., Stengel J. (2016). Mass spectrometric imaging of flavonoid glycosides and biflavonoids in *Ginkgo biloba* L.. Phytochemistry.

[16] Hasler A., Sticher O., Meier B. (1992). Identification and determination of the flavonoids from *Ginkgo biloba* by high-performance liquid chromatography. J. Chromatogr. A.

[17] Weiz G., Breccia J.D., Mazzaferro L.S. (2017). Screening and quantification of the enzymatic deglycosylation of the plant flavonoid rutin by UV–visible spectrometry. Food Chem..

[18] Halbwirth H. (2010). The creation and physiological relevance of divergent hydroxylation patterns in the flavonoid pathway. Int. J. Mol. Sci..

[19] Cunha V.R.R., Constantino V.R.L., Ando R.A. (2012). Raman spectroscopy and DFT calculations of *para*-coumaric acid and its deprotonated species. Vib. Spectrosc..

[20] Amat A., Clementi C., Miliani C., Romani A., Sgamellotti A., Fantacci S. (2010). Complexation of apigenin and luteolin in weld lake: a DFT/TDDFT investigation. Phys. Chem. Chem. Phys..

[21] Zu Y., Li C., Fu Y., Zhao C. (2006). Simultaneous determination of catechin, rutin, quercetin kaempferol and isorhamnetin in the extract of sea buckthorn (*Hippophae rhamnoides* L.) leaves by RP-HPLC with DAD. J. Pharm. Biomed. Anal..

[22] Zhou A., Sadik O.A. (2008). Comparative analysis of quercetin oxidation by electrochemical, enzymatic, autoxidation, and free radical generation techniques: a mechanistic study. J. Agric. Food Chem..

[23] Gonzales G.B., Van Camp J., Vissenaekens H., Raes K., Smagghe G., Grootaert C. (2015). Review on the use of cell cultures to study metabolism, transport, and accumulation of flavonoids: from mono-cultures to co-culture systems. Compr. Rev. Food Sci. Food Saf..

[24] Yang Y., Oh J.-M., Heo P., Shin J.Y., Kong B., Shin J., Lee J.-C., Oh J.S., Park K.W., Lee C.H. (2013). Polyphenols differentially inhibit degranulation of distinct subsets of vesicles in mast cells by specific interaction with granule-type-dependent SNARE complexes. Biochem. J..

[25] Kim D.-O., Lee C.Y. (2004). Comprehensive study on vitamin C equivalent antioxidant capacity (VCEAC) of various polyphenolics in scavenging a free radical and its structural relationship. Crit. Rev. Food Sci. Nutr..

[26] Terao J., Murota K., Kawai Y. (2011). Conjugated quercetin glucuronides as bioactive metabolites and precursors of aglycone *in vivo*. Food Funct..

[27] Moro C., Palacios I., Lozano M., D’Arrigo M., Guillamón E., Villares A., Martínez J.A., García-Lafuente A. (2012). Anti-inflammatory activity of methanolic extracts from edible mushrooms in LPS activated RAW 264.7 macrophages. Food Chem..

[28] Lin W.-W., Karin M. (2007). A cytokine-mediated link between innate immunity, inflammation, and cancer. J. Clin. Investig..

[29] Wang J., Fang X., Ge L., Cao F., Zhao L., Wang Z., Xiao W. (2018). Antitumor, antioxidant and anti-inflammatory activities of kaempferol and its corresponding glycosides and the enzymatic preparation of kaempferol. PLoS ONE.

[30] Francisco V., Figueirinha A., Costa G., Liberal J., Lopes M.C., García-Rodríguez C., Geraldes C.F.G.C., Cruz M.T., Batista M.T. (2014). Chemical characterization and anti-inflammatory activity of luteolin glycosides isolated from lemongrass. J. Funct. Food.

[31] Kim H.K., Cheon B.S., Kim Y.H., Kim S.Y., Kim H.P. (1999). Effects of naturally occurring flavonoids on nitric oxide production in the macrophage cell line RAW 264.7 and their structure–activity relationships. Biochem. Pharmacol..

[32] Kim A.-R., Shin T.-S., Lee M.-S., Park J.-Y., Park K.-E., Yoon N.-Y., Kim J.-S., Choi J.-S., Jang B.-C., Byun D.-S. (2009). Isolation and identification of phlorotannins from *Ecklonia stolonifera* with antioxidant and anti-inflammatory properties. J. Agric. Food Chem..

[33] Ko K.Y., Jeon H.L., Kim J., Kim T.S., Hong Y.-H., Jeong M.K., Park K.-H., Kim B.-H., Park S., Jang W.-H. (2020). Two tiered approaches combining alternative test methods and minimizing the use of reconstructed human cornea-like epithelium tests for the evaluation of eye irritation potency of test chemicals. Toxicol. In Vitro.

[34] McKenzie B., Kay G., Matthews K.H., Knott R.M., Cairns D. (2015). The hen’s egg chorioallantoic membrane (HET-CAM) test to predict the ophthalmic irritation potential of a cysteamine-containing gel: Quantification using Photoshop^®^ and ImageJ. Int. J. Pharm..

[35] NIH (2010). ICCVAM-Recommended Test Method Protocol: Hen’s Egg Test–Chorioallantoic Membrane (HET-CAM) Test Method.

[36] Lee B.H., Nam T.G., Kim S.Y., Chun O.K., Kim D.-O. (2019). Estimated daily per capita intakes of phenolics and antioxidants from coffee in the Korean diet. Food Sci. Biotechnol..

[37] Wolfe K.L., Liu R.H. (2007). Cellular antioxidant activity (CAA) assay for assessing antioxidants, foods, and dietary supplements. J. Agric. Food Chem..

[38] Green L.C., Wagner D.A., Glogowski J., Skipper P.L., Wishnok J.S., Tannenbaum S.R. (1982). Analysis of nitrate, nitrite, and [^15^N] nitrate in biological fluids. Anal. Biochem..

