# Supplementary material for: Characterization of Ginkgo biloba Leaf Flavonoids as Neuroexocytosis Regulators
Source: Molecules. 2020 Apr 17;25(8):1829. doi: 10.3390/molecules25081829 (PMC7221681; doi:10.3390/molecules25081829)
Supplement: Supplementary file 1 [file molecules-25-01829-s001.pdf]

molecules

MDPI

Article

Characterization of *Ginkgo Biloba* Leaf Flavonoids as Neuroexocytosis Regulators

Choongjin Ban [1,†], Joon-Bum Park [2,†], Sora Cho [3], Hye Rin Kim [2], Yong Joon Kim [2], Hyungjin Bae [4], Chinhan Kim [4], Hakhee Kang [4], Davin Jang [5], Yong Sub Shin [5], Dae-Ok Kim [5,6,*], Hyunggun Kim [7,*] and Dae-Hyuk Kweon [1,2,3,*]

[1] Institute of Biomolecule Control and Institute of Biologics, Sungkyunkwan University, Suwon 16419, Gyeonggi, Republic of Korea; pahncj@skku.edu (C.B.)
[2] Department of Integrative Biotechnology, Sungkyunkwan University, Suwon, Gyeonggi 16419, Republic of Korea; hybrik@skku.edu (J.-B.P.); hryrin@gmail.com (H.R.K.)
[3] Interdisciplinary Program in BioCosmetics, Sungkyunkwan University, Suwon 16419, Gyeonggi, Republic of Korea; jsora7254@gmail.com (S.C.); vgbk491@skku.edu (Y.J.K.)
[4] C&I lab, Kolmar Korea Co., Ltd., Seoul 06792, Republic of Korea; hjbae@kolmar.co.kr (H.B.); kimch@kolmar.co.kr (C.K.); hhkang@kolmar.co.kr (H.Kang.)
[5] Graduate School of Biotechnology, Kyung Hee University, Yongin 17104, Gyeonggi, Republic of Korea; davin1031@khu.ac.kr (D.J.); zipw4234@naver.com (Y.S.S.)
[6] Department of Food Science and Biotechnology, Kyung Hee University, Yongin 17104, Gyeonggi, Republic of Korea
[7] Department of Biomechatronic Engineering, Sungkyunkwan University, Suwon 16419, Gyeonggi, Republic of Korea
* Correspondence: DOKIM05@khu.ac.kr (D.-O.K.); hkim.bme@skku.edu (H.Kim); dhkweon@skku.edu (D.-H.K.); Tel.: +82-31-201-3796 (D.-O.K.); +82-31-290-7821 (H.Kim); +82-31-299-4850 (D.-H.K.)
† These authors contributed equally to this work.

1. *Determination of Partition Coefficient*

The logarithm of the 1-octanol/water partition coefficient (log *P*) was examined to quantify the hydrophobicity or hydrophilicity of NI, AI, Q, and K using the pre-introduced method with some modifications [1]. Two hundred microliters of NI, AI, Q, and K (5 mg·mL^{-1}) in methanol were transferred into 2-mL micro-tubes, and methanol was evaporated under a stream of nitrogen gas. After addition of 1-octanol (500 μL), the micro-tubes were vortexed for 1 min and placed in a bath-type sonicator for 5 min, and then, 500 μL DDW was added to the tubes. The mixture solution in the tubes was vortexed for 5 min and phase-separated by centrifugation (10 min, 13000 RCF). The upper (1-octanol) and lower (DDW) layers of 40 and 100 μL were collected and diluted with 1.96 mL 1-octanol and 100 μL DMSO, respectively. The solutes (NI, AI, Q, and K) dissolved in the upper and lower layers were quantified using the standard curves obtained from the UV/visible-light absorptive spectra. Finally, the log *P* values were calculated as $\log(c_{\text{octanol}}/c_{\text{DDW}})$, where c_{octanol} and c_{DDW} are the concentrations of the solutes (NI, AI, Q, and K) in the upper and lower layers, respectively.

2. *Identification of the Ginkgo Biloba Leaf Isolates*

The ^{1}H-NMR spectra of the compounds dissolved in methanol (25 mg·mL^{-1}) were obtained using a Varian Unity INOVA 500 MHz NMR spectrometer (Agilent Technologies, Santa Clara, CA, USA). The ^{1}H-NMR spectra of the GBL NI, GBL AI, Q, and K were obtained (Figure S1). In the spectrum for Q (Figure S1a), five specific peak multiplets were detected at chemical shifts (500 MHz, methanol): δ 6.17 (d, 1, *J* = 2.05 Hz, H6), 6.37 (d, 1, *J* = 2.05 Hz, H8), 6.87 (d, 1, *J* = 8.51 Hz, H5′), 7.62 (dd, 1, *J* = 8.51, 2.35 Hz, H6′), and 7.73 (d, 1, *J* = 2.35 Hz, H2′) [2]. In the spectrum for K (Figure S1b), four specific peak multiplets were detected (500 MHz, methanol): δ 6.16 (d, 1, *J* = 2.05 Hz, H6), 6.36 (d, 1, *J* = 2.05 Hz, H8), 6.90 (m, 2, H3′ and H5′), and 8.08 (m, 2, H2′ and H6′). Spectra of the GBL NI showed many peaks

in the δ, ranging from 0.5 to 5.5 ppm, corresponding to the proton resonance signals in the sugars of the flavonoid glycosides and the residuals (Figure S1c) [3]. Several other specific peak multiplets were also detected (500 MHz, methanol): δ 6.19 (m, 2), 6.39 (m, 2), 6.88 (m, 3), 7.13 (m, 1), 7.37 (m, 1), 7.48 (m, 1), 7.64 (m, 1), 7.72 (d, 1, J = 2.05 Hz), and 8.07 (m, 2). Among these proton resonance signals, the multiplets at 6.19, 6.39, 6.88, 7.64, 7.72, and 8.07 ppm correspond to the proton resonates in both aglycones and glycosides of Q and K as follows: H6 (Q/K), H8 (Q/K), H5′ (Q/K) and H3′ (K), H6′ (Q), H2′ (Q), and H2′ and H6′ (K), respectively.

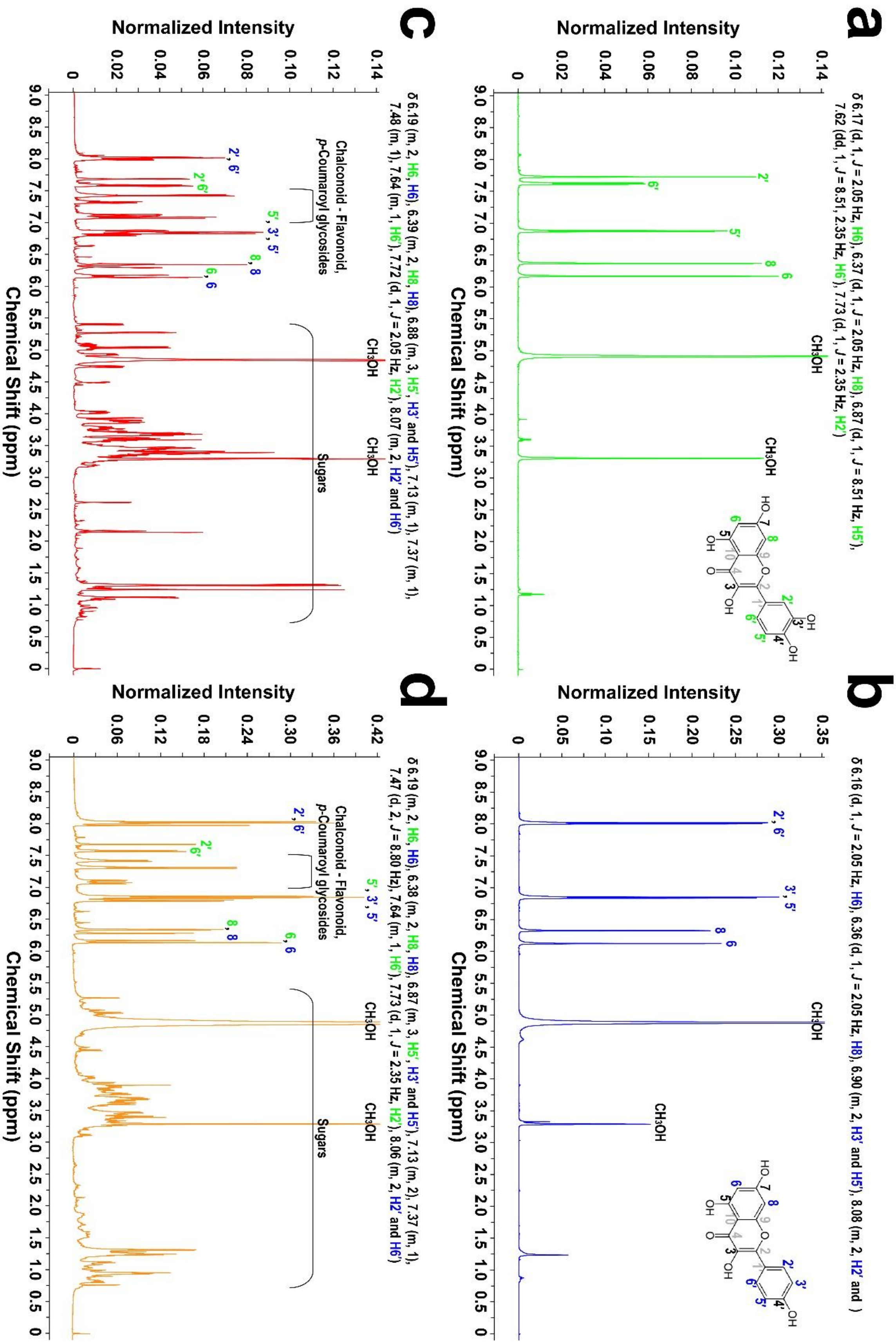

Figure S1. NMR analysis of the GBL extract components. ^{1}H-NMR spectra of (a) quercetin, (b) kaempferol, (c) GBL NI, and (d) GBL AI.

Spectra of the GBL AI showed many proton resonance signals for sugars and residuals in the δ range of 0.5–5.5 ppm, similar to those in the GBL NI spectrum. However, the intensity ratio of

aglycone and sugar for the GBL AI was smaller than that for the GBL NI, implying hydrolysis of the flavonoid glycoside sugar during the acid-isolation procedure for the GBL AI. In addition, other specific peak multiplets were detected (500 MHz, methanol): δ 6.19 (m, 2), 6.38 (m, 2), 6.87 (m, 3), 7.13 (m, 2), 7.37 (m, 1), 7.47 (d, 2, *J* = 8.80 Hz), 7.64 (m, 1), 7.73 (d, 1, *J* = 2.35 Hz, 1 H), and 8.06 (m, 2). Among these proton resonance signals, the multiplets at 6.19, 6.38, 6.87, 7.64, 7.72, and 8.06 ppm correspond to the proton resonates in both aglycones and glycosides of Q and K as follows: H6 (Q/K), H8 (Q/K), H5′ (Q/K) and H3′ (K), H6′ (Q), H2′ (Q), and H2′ and H6′ (K), respectively.

Biflavonoids, including bilobetin, ginkgetin, isoginkgetin, and sciadopitysin, are commonly found in GBL and the full extracts [4]. In a biflavonoid, C5′ of a flavonoid is generally linked with C8 of another flavonoid. According to the data obtained from the mass spectra and ^{1}H-NMR spectra, the GBL NI and GBL AI contained aglycones and glycosides of apigenin, K, luteolin, Q, and isorhamnetin. In this regard, the molecule with MW of 572.6, observed in the mass spectrum of the GBL NI (Figure 1b), is possibly the biflavonoids of those aglycones. Potential biflavonoids with MW of 572.6 might be composed of flavonoids (Q, K, taxifolin, aromadendrin, luteolin, apigenin, eriodictyol, and naringenin) and chalconoids (eriodictyol chalcone, and naringenin chalcone). The δs (ppm) for the proposed chemical structures are shown in Table S1, based on the ^{1}H-NMR spectra obtained in this study and previous studies [5–7].

Table S1. The expected ^{1}H chemical shifts (δ) of the proposed chemical structures (a and b) flavonoid-flavonoid, (c) chalconoid-flavonoid, and (d) flavonoid-chalconoid. [1]

**Position**	**δ of ^{1}H (ppm)**					
	**(a)**					
	**N-Q**	**AD-K**	**AD-L**	**E-K**	**E-L**	**T-A**
R_1	-H	-H	-H	-OH	-OH	-OH
R_2	-H	-OH	-OH	-H	-H	-OH
R_3	-OH	-H	-OH	-H	-OH	-H
R_4	-OH	-OH	-H	-OH	-H	-H
H2	5.43	5.04	5.04	5.29	5.29	4.90
H3	2.67, 3.28	4.57	4.57	2.71, 3.08	2.71, 3.08	4.49
H6	5.87	5.85	5.85	5.89	5.89	5.87
H8	5.87	5.90	5.90	5.91	5.91	5.87
H2′	>7.30	>7.30	>7.30	>6.93	>6.93	>6.95
H3′	>6.78	>6.77	>6.77			
H6′	>7.30	>7.30	>7.30	>6.93	>6.93	>6.95
H3″			6.73		6.73	6.77
H6″	>6.17	>6.16	>6.21	>6.16	>6.21	>6.16
H2‴	7.73	8.08	7.48	8.08	7.48	7.73
H3‴		6.90		6.90		6.84
H5‴	6.87	6.90	6.83	6.90	6.83	6.84
H6‴	7.62	8.08	7.19	8.08	7.19	7.73
	**(b)**					
	Q-N	K-AD	L-AD	K-E	L-E	A-T
R_1	-OH	-H	-OH	-H	-OH	-H

R_2	-OH	-OH	-H	-OH	-H	-H
R_3	-H	-H	-H	-OH	-OH	-OH
R_4	-H	-OH	-OH	-H	-H	-OH
H3			6.73		6.73	6.77
H6	6.17	6.16	6.21	6.16	6.21	6.16
H8	6.37	6.36	6.57	6.36	6.57	6.51
H2′	>7.73	>8.08	>7.48	>8.08	>7.48	>7.73
H3′		>6.90		>6.90		>6.84
H6′	>7.73	>8.08	>7.48	>8.08	>7.48	>7.73
H2″	5.43	5.04	5.04	5.29	5.29	4.90
H3″	2.67, 3.28	4.57	4.57	2.71, 3.08	2.71, 3.08	4.49
H6″	>5.87	>5.85	>5.85	>5.89	>5.89	>5.87
H2‴	7.30	7.30	7.30	6.93	6.93	6.95
H3‴	6.78	6.77	6.77			
H5‴	6.78	6.77	6.77	6.80	6.80	6.79
H6‴	7.30	7.30	7.30	6.80	6.80	6.83

	**(c)**			**(d)**		
	NC-Q	EC-K	EC-L	Q-NC	K-EC	L-EC
R_1	-H	-OH	-OH	-OH	-H	-OH
R_2	-H	-H	-H	-OH	-OH	-H
R_3	-OH	-H	-OH	-H	-OH	-OH
R_4	-OH	-OH	-H	-H	-H	-H
H2	>7.49	>7.10	>7.10			
H3	>6.81					6.73
H6	>7.49	>7.10	>7.10	6.17	6.16	6.21
H8				6.37	6.36	6.57
Hα	8.07	8.03	8.03			
Hβ	7.69	7.63	7.63			
H2′				>7.73	>8.08	>7.48
H3′	5.84	5.84	5.84		>6.90	
H5′	5.84	5.84	5.84			
H6′				>7.73	>8.08	>7.48
H2″				7.49	7.10	7.10
H3″			6.73	6.81		
H5‴				6.81	6.79	6.79
H6″	>6.17	>6.16	>6.21	7.49	6.98	6.98
Hα				8.07	8.03	8.03
Hβ				7.69	7.63	7.63

H2‴	7.73	8.08	7.48			
H3‴		6.90				
H5‴	6.87	6.90	6.83	>5.84	>5.84	>5.84
H6‴	7.62	8.08	7.19			

[1] Abbreviation: N, naringenin; Q, quercetin; AD, aromadendrin; K, kaempferol; L, luteolin; E, eriodictyol; T, taxifolin; A, apigenin; NC, naringenin chalcone; and EC, eriodictyol chalcone.

For instance, an amentoflavone is composed of two apigenins with a linkage between the B- and A′-rings, i.e., H3′ and H8″. The δ of each proton in the B- and A′-rings is larger than that of the corresponding proton in the B′- and A-rings, respectively, because of the larger resonance effect by the linkage between two aromatic rings [8]. In contrast, there was little difference in the between the C- and C′-rings. Chemical shifts δ (ppm) were 6.45 (H8) and 6.18 (H6) in the A-ring; 8.02 (H2′), 8.00 (H6′), 7.12 (H5′) in the B-ring; 6.70 (H3) in the C-ring; 6.37 (H6″) in the A′-ring; 7.58 (H2‴ and H6‴) and 6.80 (H3‴ and H5‴) in the B′-ring; and 6.70 (H3″) in the C′-ring. This tendency is applicable to the δs of protons in the proposed chemical structures. As a result, various δ values are feasible for the structures, as observed in Table S1. Particularly, the δ multiplets at 7.13, 7.37, and 7.48 ppm in both ^{1}H-NMR spectra for NI and AI that did not correspond with those for the abundant flavonoids in the GBL (apigenin, K, luteolin, Q, and isorhamnetin), can thereby correspond with those for the proposed structures. Besides, other compounds corresponding with these δ multiplets (7.13, 7.37, and 7.48 ppm) can be *p*-coumaric esters (Figure S2e) [9], potentially found in the GBL (Table S2). To summarize, Q and K were abundant in NI and AI. The apigenin, luteolin, isorhamnetin, and other compounds, including biflavonoids, chalconoid-flavonoid, flavonoid-chalconoid, and *p*-coumaroyl flavonoid glycosides were rarely found. While most of the flavonoids in the NI were present as glycosides, the majority and minority of the flavonoids in the AI were aglycones and glycosides, respectively, owing to acidolyzing isolation.

Table S2. The expected ^{1}H chemical shift (δ) of coumaric acid.

**Position**	**δ of ^{1}H (ppm)**
H2	7.53
H3	6.82
H5	6.82
H6	7.54
H7	7.53
H8	6.32

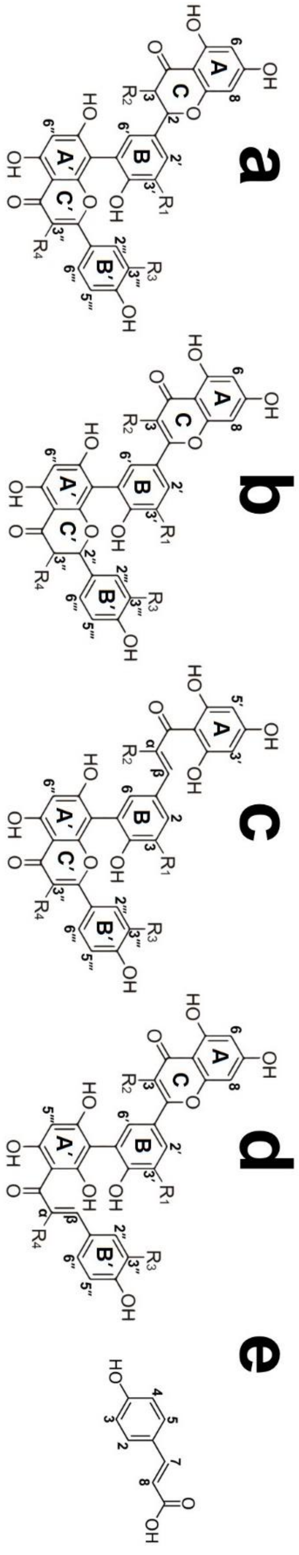

Figure S2. Proposed chemical structures of the GBL extracts. (a) and (b) Flavonoid-flavonoid, (c) chalconoid-flavonoid, and (d) flavonoid-chalconoid, possibly present in the *Ginkgo biloba* leaf isolates, based on the literature and data obtained in the HPLC-MS/MS and ^{1}H-NMR measurements (A, B, C, A′, B′, and C′: A-, B-, C-, A′-, B′-, and C′-rings). Chemical structure of (e) coumaric acid.

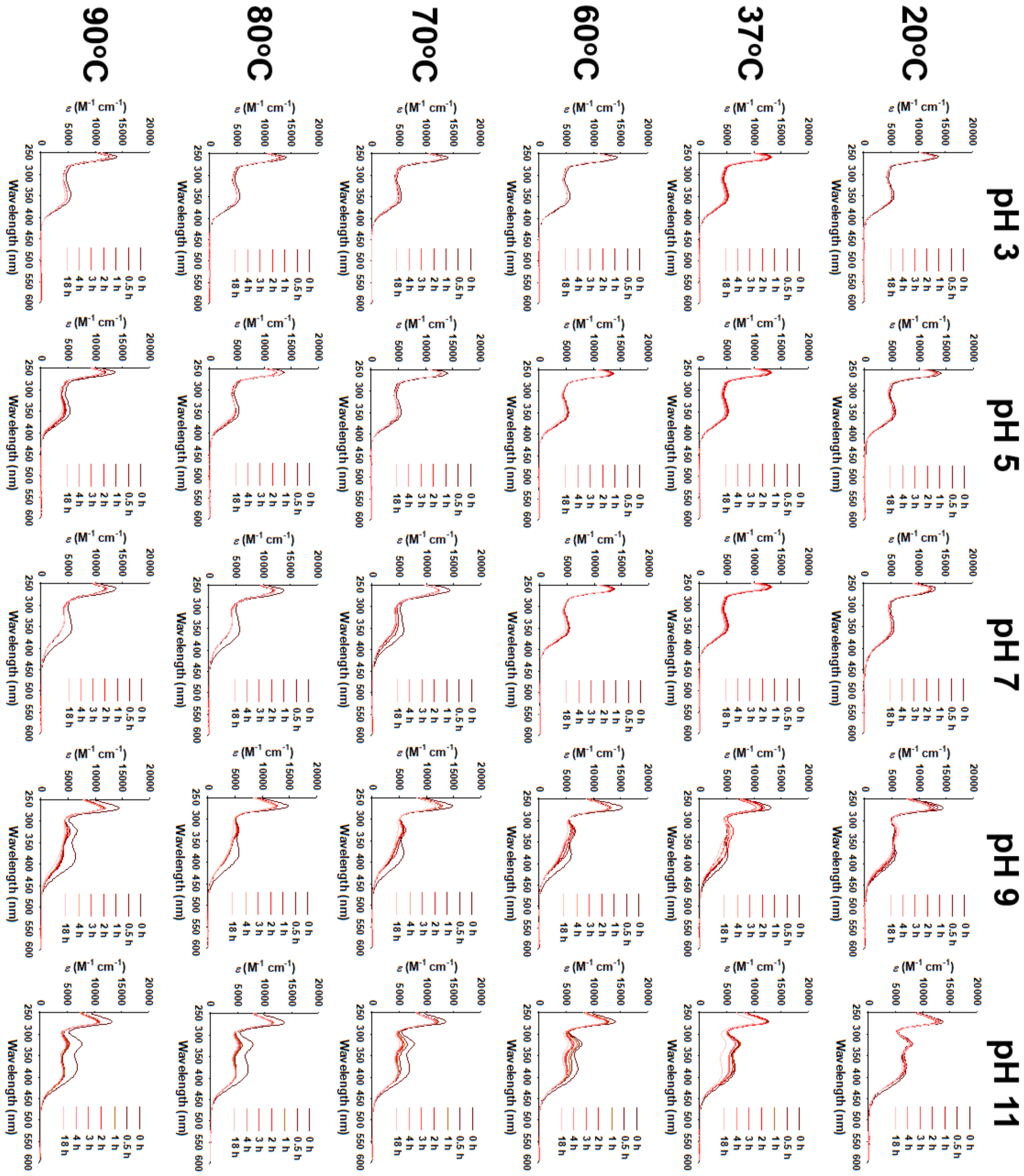

Figure S3. Changes in the UV/visible absorption spectra of the GBL NI in aqueous solution at pH 3–11 and 20–90 °C.

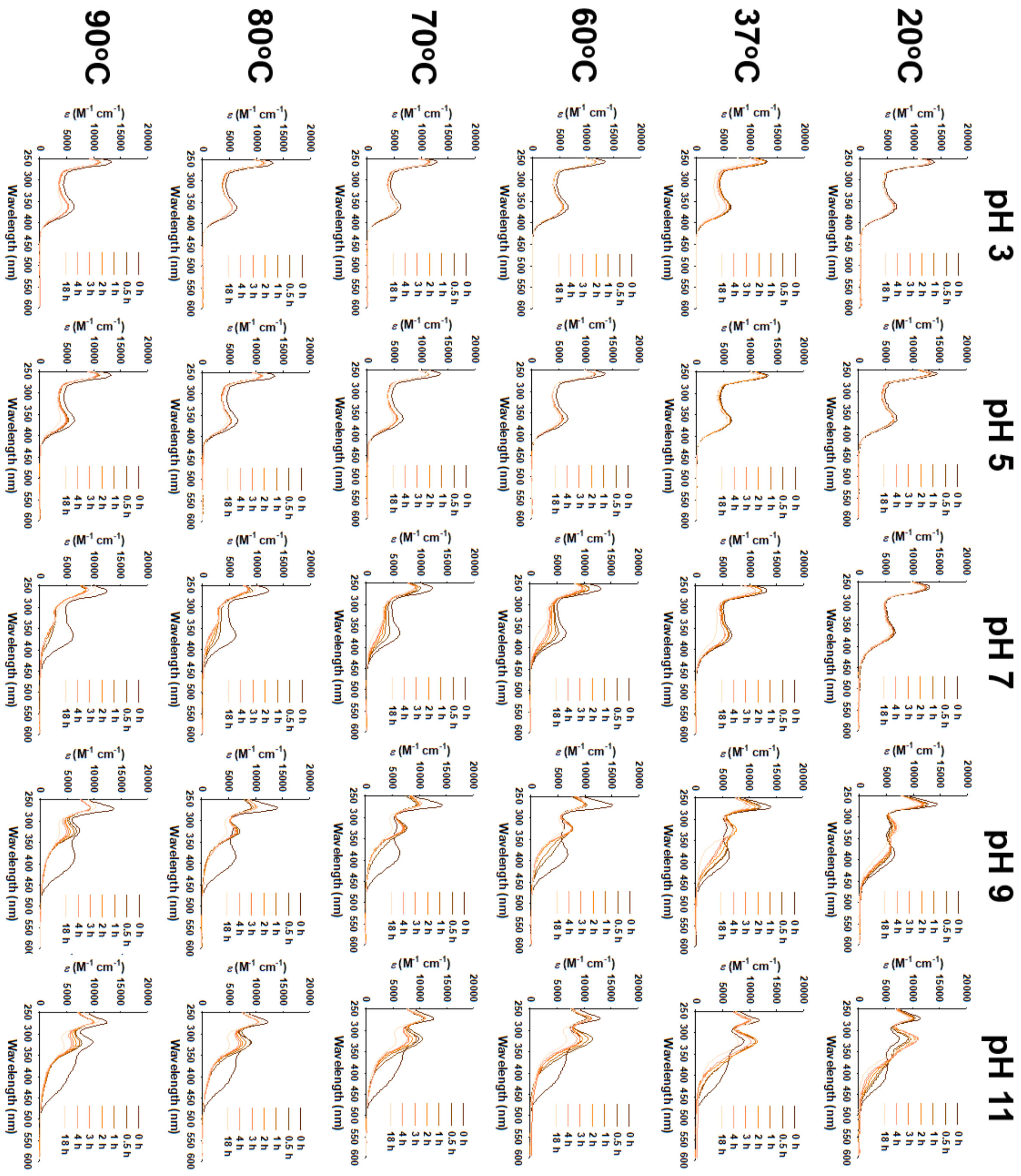

Figure S4. Changes in the UV/visible absorption spectra of the GBL AI in aqueous solution at pH 3–11 and 20–90 °C.

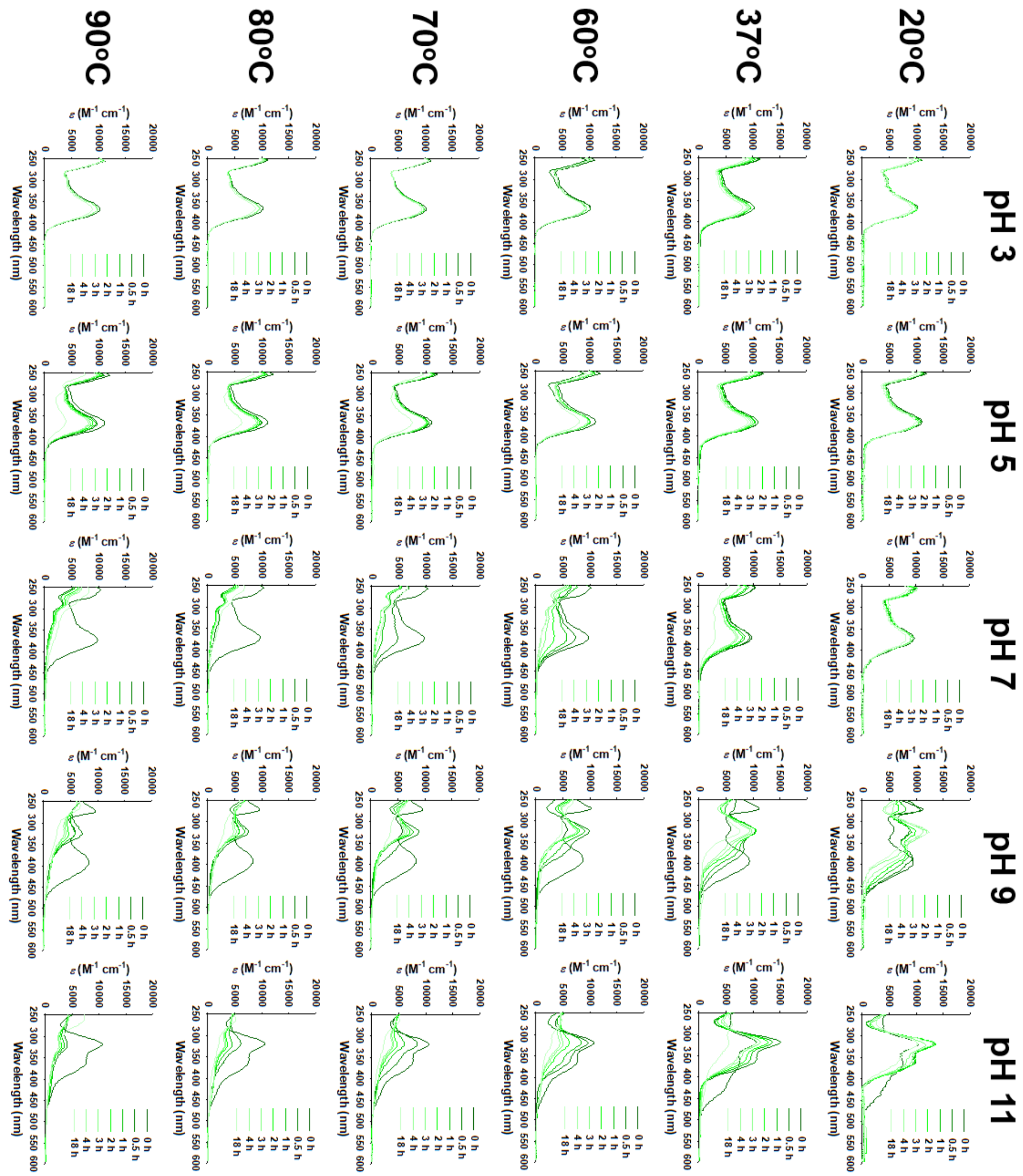

Figure S5. Change in the UV/visible absorption spectra of quercetin in aqueous solution at pH 3–11 and 20–90 °C.

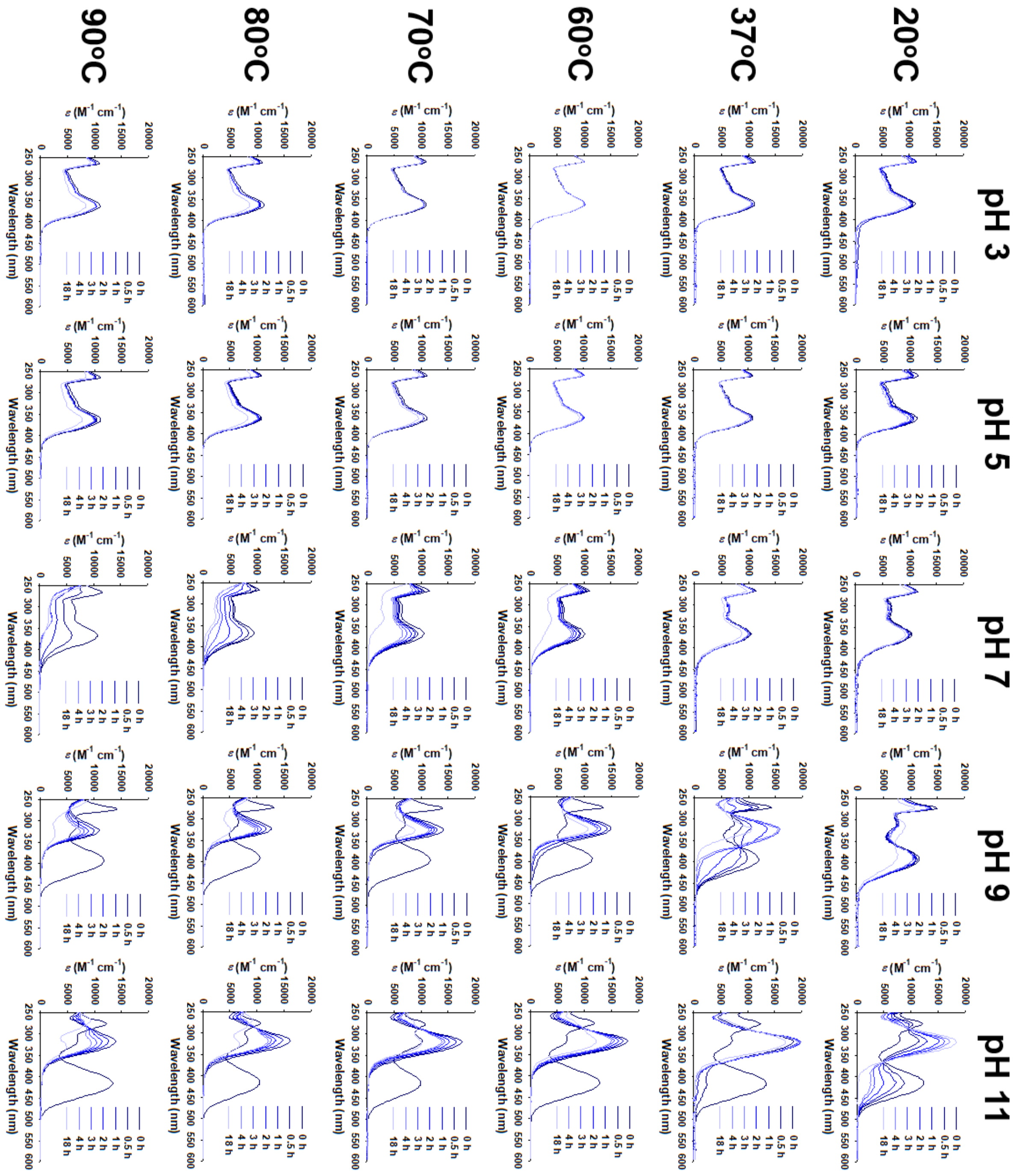

Figure S6. Changes in the UV/visible absorption spectra of kaempferol in aqueous solution at pH 3–11 and 20–90 °C.

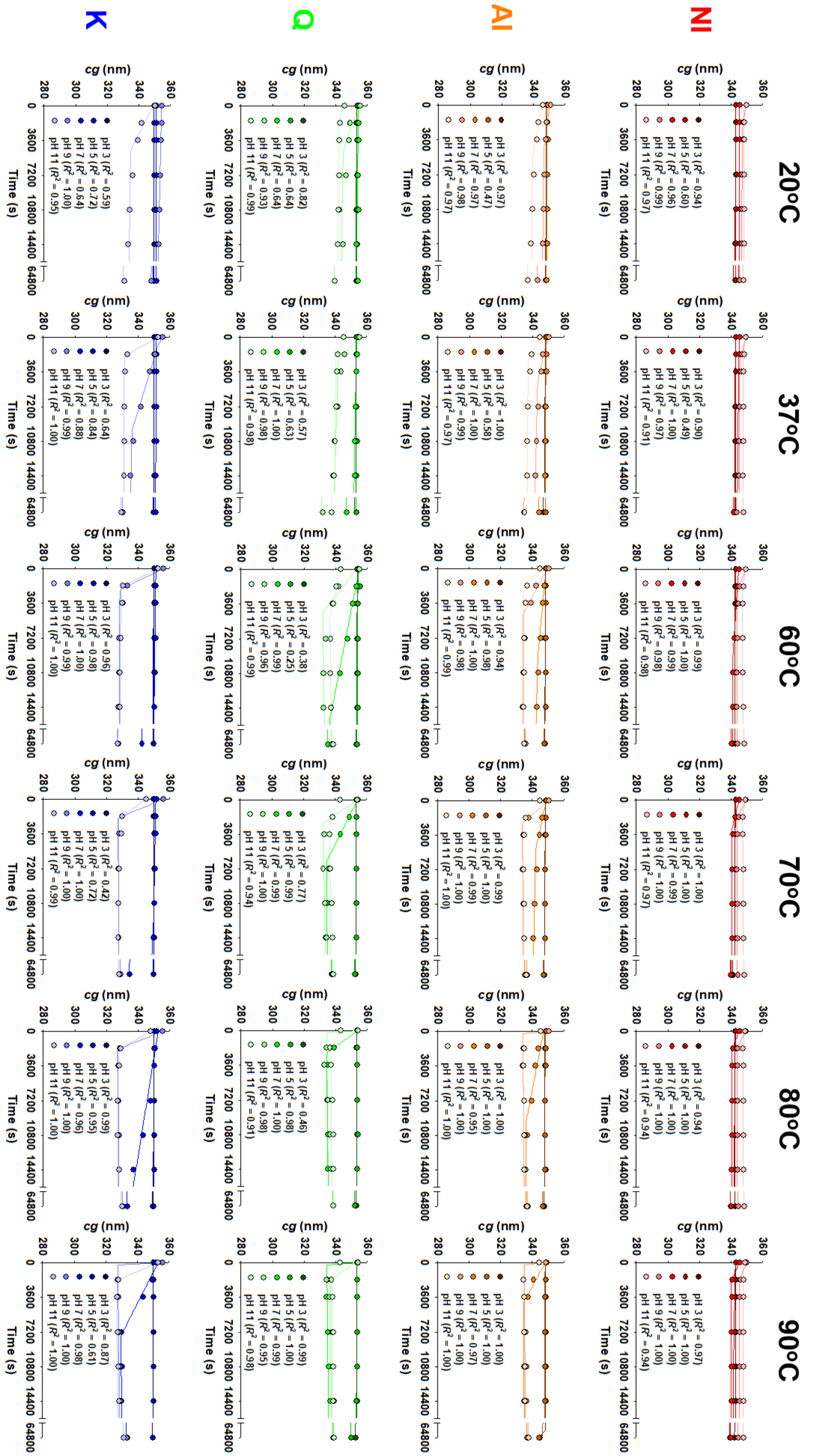
NI
AI
Q
K
20°C
37°C
60°C
70°C
80°C
90°C
cg (nm)
Time (s)

Figure S7. Changes in the center of gravity [$cg = \sum \varepsilon_i \lambda_i / \sum \varepsilon_i$; ε_i , absorptivity at a wavelength range (λ_i, 300–400nm)] in the UV/visible-light absorptive spectra of the GBL NI, GBL AI, quercetin (Q), and kaempferol (K) during incubation at the designated conditions. The cg was determined using the two segment linear fitting curves [$cg = k_1 t + cg_1 \ (0 \le t < t_1) = k_2 t + cg_2 \ (t_1 \le t)$].

Table S3. Significantly different levels of the cg shifting rate in the UV/visible-light absorptive spectra of the GBL isolates under the Tukey's test ($n = 3$; $P < 0.05$).

pH	Temperature (°C)	Samples	Significant different levels (1–13)
3	20	NI	13
		AI	13
		Q	13
		K	13
	37	NI	13
		AI	13
		Q	13
		K	13
	60	NI	13
		AI	13
		Q	13
		K	13
	70	NI	13
		AI	13
		Q	13
		K	13
	80	NI	13
		AI	13
		Q	13
		K	13
	90	NI	13
		AI	13
		Q	13
		K	13
5	20	NI	13
		AI	13
		Q	13
		K	13
	37	NI	13
		AI	13
		Q	13
		K	13
	60	NI	13
		AI	13

		Q	13
		K	13
	70	NI	13
		AI	13
		Q	13
		K	13
	80	NI	13
		AI	13
		Q	13
		K	13
	90	NI	13
		AI	13
		Q	13
		K	13
7	20	NI	13
		AI	13
		Q	13
		K	13
	37	NI	13
		AI	13
		Q	12–13
		K	13
	60	NI	12–13
		AI	12–13
		Q	10–13
		K	13
	70	NI	10–13
		AI	10–13
		Q	8–13
		K	13
	80	NI	10–13
		AI	10–13
		Q	3–13
		K	11–13
	90	NI	9–13
		AI	8–13
		Q	2–10
		K	8–13
9	20	NI	11–13
		AI	10–13
		Q	10–13
		K	13

	37	NI	10–13
		AI	10–13
		Q	5–13
		K	10–13
	60	NI	10–13
		AI	8–13
		Q	5–13
		K	2–8
	70	NI	10–13
		AI	3–13
		Q	3–13
		K	2–5
	80	NI	9–13
		AI	3–13
		Q	3–13
		K	2–4
	90	NI	9–13
		AI	2–7
		Q	1–2
		K	2–3
11	20	NI	12–13
		AI	11–13
		Q	10–13
		K	7–13
	37	NI	12–13
		AI	8–13
		Q	10–13
		K	3–13
	60	NI	11–13
		AI	6–13
		Q	10–13
		K	3–11
	70	NI	11–13
		AI	5–13
		Q	9–13
		K	2–10
	80	NI	11–13
		AI	4–13
		Q	3–12
		K	2–9
	90	NI	10–13
		AI	3–13

Q	1
K	2–6

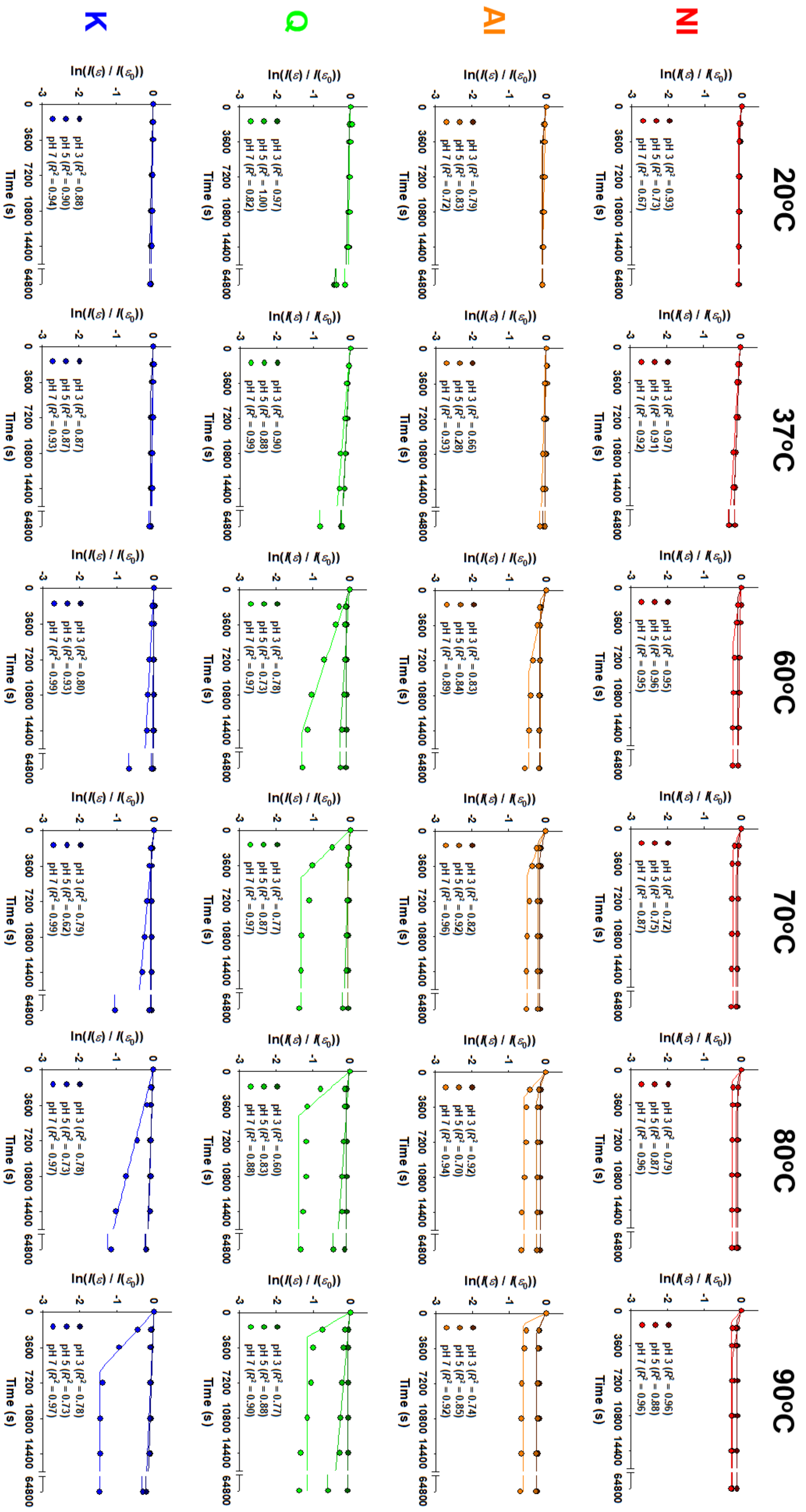
20°C
37°C
60°C
70°C
80°C
90°C
NI
AI
Q
K
ln(I(ε) / I(ε0))
Time (s)

Figure S8. Exponential decay-plateau fitting curves. Decay kinetics were fitted to the following equation from the UV/visible absorption spectra of the GBL NI, GBL AI, quercetin (Q), and kaempferol (K) during incubation at the designated conditions. $\ln(I(\varepsilon)/I(\varepsilon_0)) = -kt\ \left(0 \leq t < t_\mathrm{p}\right) = p\ \ (t_\mathrm{p} \leq t)$; $I(\varepsilon)$, integration value of the absorptivity at the incubation time (t); $I(\varepsilon_0)$, integration value of the absorptivity at the initiation of the incubation; k, rate constant value; p, constant on the plateau.

Table S4. Significantly different levels of the non-oxidized remaining amount $[I(\varepsilon)/I(\varepsilon_0)]$ in the UV/visible-light absorptive spectra of the GBL isolates under the Tukey's test (n = 3; $P < 0.05$).

pH	Temperature (°C)	Samples	Significant different levels (1–32)
3	20	NI	27–31
		AI	24–30
		Q	30–32
		K	31–32
	37	NI	26–31
		AI	18–23
		Q	29–32
		K	26–31
	60	NI	24–30
		AI	16–20
		Q	24–30
		K	24–30
	70	NI	24–30
		AI	14–17
		Q	22–29
		K	23–30
	80	NI	22–29
		AI	11–13
		Q	11–13
		K	14–16
	90	NI	19–26
		AI	10–11
		Q	8
		K	12–15
5	20	NI	32
		AI	19–24
		Q	12–15
		K	28–32
	37	NI	28–31
		AI	17–21
		Q	12–14
		K	28–31
	60	NI	23–30

		AI	16–19
		Q	11–13
		K	25–31
	70	NI	20–27
		AI	14–17
		Q	9
		K	23–30
	80	NI	19–25
		AI	12–14
		Q	8
		K	13–15
	90	NI	12–14
		AI	11–12
		Q	6–7
		K	10
7	20	NI	23–29
		AI	21–28
		Q	16–21
		K	24–30
	37	NI	15–18
		AI	10
		Q	5
		K	18–22
	60	NI	12–15
		AI	8
		Q	3–4
		K	6
	70	NI	12–14
		AI	8
		Q	2–3
		K	4
	80	NI	12–14
		AI	7
		Q	2–3
		K	2–3
	90	NI	11–13
		AI	7
		Q	1–2
		K	1

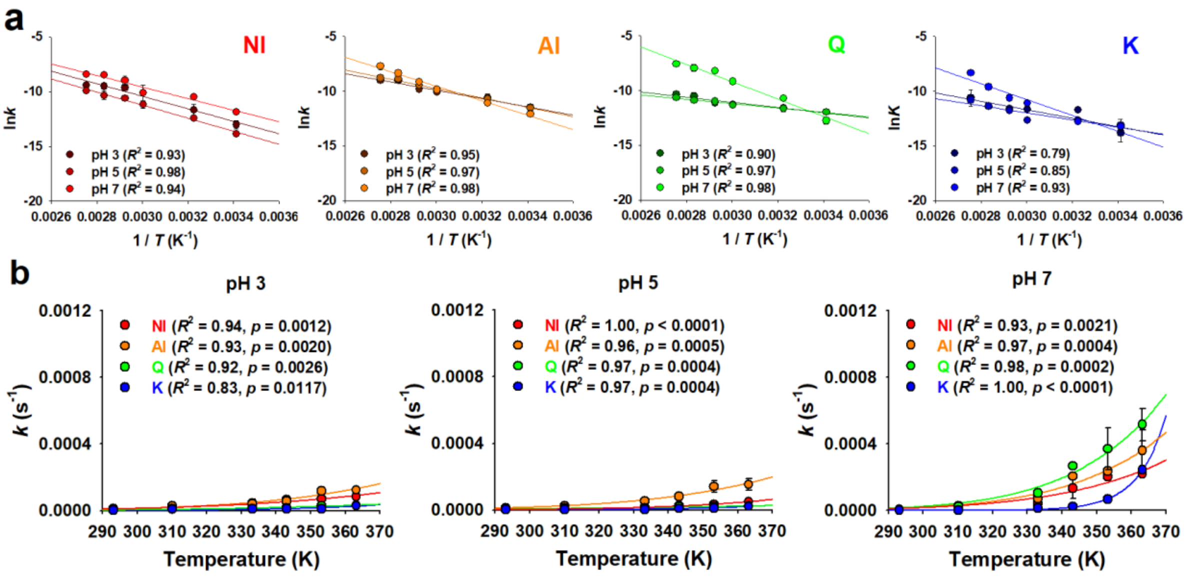

Figure S9. Decay kinetics of the GBL extracts. (a) The Arrhenius plots ($\ln k = -(E_a/R)(1/T) + \ln A$; k: decay rate constant, E_a: activation energy, R: gas constant, T: absolute temperature, A: pre-exponential factor) and (b) k values of the GBL NI, GBL AI, quercetin (Q), and kaempferol (K) in aqueous solutions at pH 3–11 and 293–363 K.

Table S5. Mobile phase composition for sample separation. The % composition of double-deionized water (DDW, 0.1% formic acid) and acetonitrile (ACN, 0.1% formic acid) in the gradient mobile phase during the sample separation. [1]

Time (min)	DDW (%)	ACN (%)	Flow rate (mL·min^{-1})
0–3	95	5	0.2
10–11	70	30	
14–17	0	100	
18–20	95	5	

[1] EC_{50}: 50% effective concentration; CC_{50}: 50% cytotoxic concentration; data with different letters in the same column represent significant differences according to the Tukey's test ($n = 3$; mean ± SD; $P < 0.05$).

References

1. Rothwell, J.A.; Day, A.J.; Morgan, M.R.A., Experimental determination of octanol−water partition coefficients of quercetin and related flavonoids. *J. Agric. Food Chem.* **2005,** *53*, 4355–4360.
2. De Souza, L.A.; Tavares, W.M.G.; Lopes, A.P.M.; Soeiro, M.M.; De Almeida, W.B., Structural analysis of flavonoids in solution through DFT ^{1}H NMR chemical shift calculations: Epigallocatechin, Kaempferol and Quercetin. *Chem. Phys. Lett.* **2017,** *676*, 46–52.
3. Le Gall, G.; Colquhoun, I.J.; Davis, A.L.; Collins, G.J.; Verhoeyen, M.E., Metabolite profiling of tomato (*Lycopersicon esculentum*) using ^{1}H NMR spectroscopy as a tool to detect potential unintended effects following a genetic modification. *J. Agric. Food Chem.* **2003,** *51*, 2447–2456.
4. van Beek, T.A.; Montoro, P., Chemical analysis and quality control of *Ginkgo biloba* leaves, extracts, and phytopharmaceuticals. *J. Chromatogr. A* **2009,** *1216*, 2002–2032.
5. Zhang, X.F.; Hung, T.M.; Phuong, P.T.; Ngoc, T.M.; Min, B.-S.; Song, K.-S.; Seong, Y.H.; Bae, K.H., Anti-inflammatory activity of flavonoids from *Populus davidiana*. *Arch. Pharmacal. Res.* **2006,** *29*, 1102–1108.
6. Le Bail, J.-C.; Pouget, C.; Fagnere, C.; Basly, J.-P.; Chulia, A.-J.; Habrioux, G., Chalcones are potent inhibitors of aromatase and 17β-hydroxysteroid dehydrogenase activities. *Life Sci.* **2001,** *68*, 751–761.
7. Xu, S.; Shang, M.-Y.; Liu, G.-X.; Xu, F.; Wang, X.; Shou, C.-C.; Cai, S.-Q., Chemical constituents from the rhizomes of *Smilax glabra* and their antimicrobial activity. *Molecules* **2013,** *18*, 5265–5287.
8. Ryu, Y.B.; Jeong, H.J.; Kim, J.H.; Kim, Y.M.; Park, J.-Y.; Kim, D.; Naguyen, T.T.H.; Park, S.-J.; Chang, J.S.; Park, K.H., Biflavonoids from *Torreya nucifera* displaying SARS-CoV 3CL^{pro} inhibition. *Bioorg. Med. Chem.* **2010,** *18*, 7940–7947.
9. Christophoridou, S.; Dais, P., Detection and quantification of phenolic compounds in olive oil by high resolution ^{1}H nuclear magnetic resonance spectroscopy. *Anal. Chim. Acta* **2009,** *633*, 283–292.

© 2020 by the authors. Submitted for possible open access publication under the terms and conditions of the Creative Commons Attribution (CC BY) license (http://creativecommons.org/licenses/by/4.0/).
